# Negative symptoms and resting state functional connectivity: Leveraging ecological momentary assessment and individual-specific techniques^☆^

**DOI:** 10.1016/j.schres.2026.02.016

**Published:** 2026-03-10

**Authors:** Nada Dalloul, Sridhar Kandala, Erin Moran, Deanna M. Barch

**Affiliations:** a Department of Psychological & Brain Sciences, Washington University, St. Louis, MO, United States of America; b Laboratory of Behavioral Neuroscience, National Institute on Aging, National Institutes of Health, Baltimore, MD, United States of America; c Department of Psychiatry, Washington University School of Medicine, St. Louis, MO, United States of America; d Department of Radiology, Mallinckrodt Institute of Radiology, Washington University School of Medicine, St. Louis, MO, United States of America

**Keywords:** Negative symptoms, Functional connectivity, Psychosis, Schizophrenia, Transdiagnostic, Ecological momentary assessment

## Abstract

There are indications that motivational deficits in psychotic and mood disorders are related to differences in functional connectivity. However, the literature is mixed, partly due to the limitations of traditional methods in accounting for individual variability. Ecological momentary assessment (EMA) and individual-specific techniques can account for such variability. By leveraging these methods, this study aims to elucidate transdiagnostic relationships between motivational deficits and resting-state functional connectivity. 144 participants (29 schizophrenia/schizoaffective; 38 bipolar, 37 major depressive, 40 controls) completed EMA on motivation and pleasure (MAP) and resting-state BOLD scans. Individual-specific network connectivity matrices were calculated with an empirically validated template-matching technique. Bayesian hierarchical regression models assessed relationships between anticipatory and consummatory MAP and within-network connectivity, participation coefficient, and number of vertices for eight networks (default mode, cingulo-opercular, dorsal attention, ventral attention, salience, fronto-parietal, dorsal somato-motor, ventral somato-motor). Analyses demonstrated intact MAP for bipolar and major depressive disorders, and elevated MAP for schizophrenia/schizoaffective disorder. Moreover, MAP was positively related to within-network connectivity of the dorsal attention network, positively related to participation coefficient of the ventral attention network in schizophrenia/schizoaffective disorder, negatively related to within-network connectivity of the salience network in schizophrenia/schizoaffective disorder, and positively related to spatial extent of the salience network in controls. Overall, the findings highlight the role of attentional processes in MAP and suggest that underlying neural mechanisms vary by diagnosis. The results emphasize the complexity of MAP deficits and underscore the need for research to consider inter-individual variability to fully understand their phenomenology and neural basis.

## 1. Introduction

Symptoms of psychotic disorders vary widely, ranging from positive symptoms, which include hallucinations, delusions, and disorganization, to negative symptoms. Negative symptoms are defined as reductions in what would be considered typical functions or daily human experiences. They can be conceptualized as two separable domains: diminished expressivity and diminished motivation and pleasure (Castonguay et al., 2021). Diminished motivation and pleasure (MAP) consists of anhedonia (decreased ability to experience pleasure) and avolition (reduced motivation to carry out goal-directed behavior). MAP symptoms are highly prevalent in psychotic disorders (An Der Heiden et al., 2016; Bobes et al., 2010; Rabinowitz et al., 2013) and have been shown to be major contributors to disability, distress, and overall life satisfaction (García-Portilla et al., 2021; Harvey, 2014; Menendez-Miranda et al., 2015; Strassnig et al., 2018). Moreover, MAP symptoms are present across mood disorders, with and without psychotic features (Bowie et al., 2010). Despite this, current treatments are ineffective at alleviating MAP symptoms. While pharmaceutical interventions generally lessen positive and disorganized symptoms, their effect on MAP symptoms are negligible (Carbon and Correll, 2014; Castonguay et al., 2021). Likewise, psychosocial approaches like Cognitive Behavioral Therapy for psychosis, social skills training, and meditation-based treatment demonstrate minimal effectiveness in reducing MAP symptoms (Sitko et al., 2020; Velthorst et al., 2015).

A potential reason for the lack of effective interventions is the unclear cognitive and neural bases for MAP symptoms. Theories propose that these symptoms may stem from deficits in reward, motivation, or higher-order cognitive processes (Moran et al., 2023). Specifically, MAP symptoms have been associated with BOLD responses in striatal regions, basal ganglia, and prefrontal cortex (Bakker et al., 2019; Culbreth et al., 2020; Kasanova et al., 2017; Moran et al., 2023). However, research investigating the specific cognitive impairments and related neural regions has yielded inconsistent findings, with many studies failing to replicate, suggesting that a crucial piece of the puzzle is missing. Preliminary, albeit limited, evidence indicates that dysconnectivity in the neural areas and networks that facilitate these cognitive functions might play a role in MAP symptoms rather than the aberrant activation of a single neural region (Correll and Schooler, 2020; McCutcheon et al., 2020). This idea aligns with the dysconnectivity theory of schizophrenia, which proposes that schizophrenia arises from disrupted functional communication and integration among various neural regions (Frangou, 2014; Friston, 1998; Pettersson-Yeo et al., 2011a; Stephan et al., 2009). The dysconnectivity theory of schizophrenia has garnered significant backing from biological (Giraldo-Chica and Woodward, 2017; Limongi et al., 2020) and genetic (Birnbaum and Weinberger, 2017) research. As a result, functional connectivity (FC) has become a promising approach for exploring the neural underpinnings of MAP symptoms.

Two categories of resting-state functional connectivity methods can be differentiated: node-based and voxel-based (Bijsterbosch, 2017). Broadly, node-based methods utilize functionally defined nodes that are typically larger than voxels but smaller than networks (Van Den Heuvel et al., 2008). While both methods have distinct advantages (Bijsterbosch, 2017; Lee et al., 2013; Power et al., 2011), prior work has bolstered the clinical utility of node-based methods and demonstrated that node-based methods allow for a detailed and comprehensive assessment of the brain’s functional organization. In contrast, while voxel-based methods provide a finer level of detail, they are at higher risk of higher false discovery rates and are limited in interpretability because voxels lack clear functional meaning (Bullmore and Sporns, 2009; Van Den Heuvel et al., 2008). To synthesize the current literature on the relationships between node-based resting-state functional connectivity and negative symptoms across psychotic and mood disorders, we conducted a recent systematic review (Dalloul et al., 2025). Despite the advantages and promise of FC, we identified that research linking node-based FC to negative symptoms is variable and limited. Nonetheless, several trends were observed. Some studies reported that negative symptoms were negatively related to functional segregation i.e., decreased short-range neural connections (Fair et al., 2008; Jiang et al., 2022; Park et al., 2017; Peeters et al., 2016; Su et al., 2015; Yu et al., 2017; Zhang et al., 2021). This suggests negative symptoms are linked to a lack of specialized brain circuits. Additionally, several found a significant relationship between general connectivity and negative symptoms, though divergent findings make directionality unclear (Doucet et al., 2020; Mellem et al., 2020; Skudlarski et al., 2010; Yu et al., 2017; Zhang et al., 2021). Similarly, while significant network-specific associations were found, conflicted findings muddle directionality. Negative symptoms were found to be related to within- and between-network connectivity of the salience network (Lee et al., 2018; Wang et al., 2020) as well as within- and between-network connectivity of the frontoparietal and sensorimotor networks (Wang et al., 2020). Kebets et al. (2019), who used a multivariate technique to identify several latent factors linking psychopathology to connectivity patterns, emphasized the significant role of the somatomotor networks in negative symptoms while also implicating the role of the visual, dorsal attention, salience, and cingulo-opercular networks. Mellem et al. (2020) utilized a fully data-driven machine learning method to identify transdiagnostic multimodal models for dysregulated mood, anhedonia, and anxiety. They determined significant relationships with no specific directionality, but that largely implicated the role of the default mode network (Mellem et al., 2020). Nevertheless, the abundance of non-significant findings restricts the interpretation of these studies, revealing methodological limitations in the existing literature.

A potentially key reason for the inconsistent findings linking FC and MAP symptoms is that traditional FC data analysis methods have significant reliability and validity concerns (Botvinik-Nezer and Wager, 2023; Caeyenberghs et al., 2024; Makowski et al., 2019; Marek et al., 2022). These methods typically depend on group-averaged data, which obscure crucial individual differences in the location and extent of functional networks (Gordon et al., 2017a; Levi et al., 2023). In contrast, individual-specific FC (IS-FC) approaches take into account inter-individual variability (i.e., variability between individuals) and have proven to be more reliable and stable (Gordon et al., 2017b). Additionally, these IS-FC techniques demonstrate stronger links to behavior than group-averaged FC metrics (Gordon et al., 2017b). For instance, research conducted by Wang et al. (2020) revealed significant correlations between general negative symptoms and individual-specific metrics, which were not observable with traditional group-averaged methods.

IS-FC approaches also allow for the investigation of the spatial extent of resting state networks. Spatial extent of resting state networks corresponds to how specific neural functions are prioritized (Mamah et al., 2024). This often overlooked aspect can provide insights into how individual differences in network size relate to individual differences in symptoms and behavior. This is a burgeoning area of research with promising results supporting the importance of functional network spatial extent in psychopathology. For example, functional network topography has been associated with polygenic risk for transdiagnostic psychopathology (Sun et al., 2024). Moreover, research has established the role of functional network size in clinical depression (Northoff, 2021), with indications of a specific increase in salience network size (Lynch et al., 2024). Regarding psychotic spectrum disorders, Mamah et al. (2024) reported that individuals with schizophrenia and psychotic experiences, on average, have larger language and dorsal attention networks and smaller fronto-parietal networks. Overall, these studies provide preliminary evidence for the importance of resting state networks’ spatial extent in psychotic and mood disorders. They add further support for using IS-FC to assess relationships with symptomatology and address the current literature’s gaps.

In addition to the limitations of FC methodology, traditional methods for assessing MAP symptoms have significant shortcomings. MAP symptoms are frequently evaluated using self-report questionnaires or clinical assessments conducted by clinicians during study visits, usually at a single time point. These lab evaluations may be limited in their ability to accurately portray a patient’s experience of symptoms in daily life (Correll and Schooler, 2020; Daniel, 2013; Lyne et al., 2018; MacDonald et al., 2006). These assessments typically require patients to reflect retrospectively on their symptoms over a specific time frame. This can be hindered by memory biases, such as having difficulty recalling more temporally distant memories or only remembering significant events (Moran et al., 2017a). Moreover, prior studies indicate that state-like factors can impact lab evaluations at just one time, and these evaluations often overlook intra-individual variability, such as daily and weekly symptom fluctuations (MacDonald et al., 2006). Recognizing this variability is crucial to fully understanding the range of MAP symptoms (Strauss and Cohen, 2017). A potential solution to these challenges is ecological momentary assessment (EMA), which enables researchers to gather data on negative symptoms in participants’ everyday environments. This approach enhances the ecological validity of symptom data and captures significant trends over time, which may improve patient symptom assessment and management (Firth and Torous, 2015).

Though MAP symptoms are typically conceptualized as symptoms of psychotic disorders, they are also present in mood disorders and affect daily functioning (Bowie et al., 2010). Understanding the transdiagnostic nature of these deficits can inform interventions that benefit both psychotic and mood disorders, including major depressive disorder and bipolar disorder with psychotic features. Despite the transdiagnostic nature of these symptoms, there has been limited transdiagnostic research focusing on node-based functional connectivity and negative symptoms. While several studies have included participants with bipolar disorder with psychosis, they failed to explore the transdiagnostic nature of these symptoms. These studies often included a negligible sample of patients diagnosed with bipolar disorder, did not clarify the presence of psychotic features, neglected to incorporate diagnosis as a predictor or analyzed data separately by diagnosis (Baker et al., 2019; Kebets et al., 2019; Mamah et al., 2013; Mellem et al., 2020; Moser et al., 2018). Furthermore, transdiagnostic research including major depressive disorder remains absent. This gap is significant, as determining whether shared mechanisms exist across disorders could greatly influence treatment strategies.

This study focuses on examining the connections between MAP symptoms and FC by utilizing advanced techniques that enhance the validity of these relationships — IS-FC and EMA. Accordingly, this study aims to identify whether within- and between-network connectivity or spatial extent of eight resting state networks (default mode, cingulo-opercular, dorsal attention, ventral attention, salience, fronto-parietal, dorsal somato-motor, and ventral somato-motor) predicts current or anticipatory motivation and pleasure as assessed by EMA across individuals with psychotic and mood disorders. Based on prior research, we predict that MAP symptoms will be related to connectivity and spatial extent of the default-mode, cingulo-opercular, dorsal attention, ventral attention, salience, and fronto-parietal networks. Furthermore, we expect that these relationships will be largely transdiagnostic. This research has the potential to fulfill the critical need to develop effective, personalized, and transdiagnostic clinical interventions for MAP symptoms.

## 2. Method

### 2.1. Participants

Participants were recruited through treatment centers and community outreach. They included community controls (CON) or individuals diagnosed with schizophrenia/schizoaffective disorder (SZ), bipolar disorder with psychosis (BD), or major depressive disorder (MDD). A master’s or Ph.D.-level clinician confirmed the participants’ diagnostic status, including a history of psychosis for the BD individuals, using the Structured Clinical Interview for DSM-5 (First, 2015). Clinician-rated assessments, i.e., CAINS (Forbes et al., 2010) and BPRS (Overall and Gorham, 1962), and self-report assessments, MAP-SR (Llerena et al., 2013) and CES-D-10 (Roberts and Vernon, 1983), were completed in the first lab visit. In total, complete data from 166 participants were collected. Data from 18 participants were excluded after the resting-state data cleaning process, and data from 4 participants were excluded following the EMA data cleaning process (see below for details). Ultimately, data from 144 participants were included in the analyses. This sample comprised 29 SZ, 37 MDD, 38 BD, and 40 CON. Exclusion criteria included a DSM-5 diagnosis of substance abuse or dependence within the past 6 months, an IQ below 70 as measured by the Wechsler Test of Adult Reading (Wechsler, 2001), or a history of severe head trauma and/or loss of consciousness. Additional exclusion criteria for patient groups included medication changes within the month prior to study participation and inpatient or partial hospital status. Exclusion criteria for controls also included no current or past major depression, no current use of psychotropic medication, and no personal history or immediate family history of schizophrenia, schizoaffective disorder, or bipolar disorder. All participants provided written informed consent to the protocol approved by the Washington University Institutional Review Board.

### 2.2. Procedure

#### 2.2.1. Ecological Momentary Assessment (EMA)

As part of the EMA protocol, participants were prompted on their smartphones four times daily, between 9 am and 9 pm, at semi-fixed intervals for 14 days. They were asked to rate their current and anticipated motivation and pleasure for social and non-social activities on a five-point Likert scale (Table 1). To ensure data quality and as per previous studies (Moran et al., 2017b, 2019, 2024), we included data from participants who responded to at least 33% of the prompts in the analyses, which resulted in the exclusion of 4 participants (2 CON, 1 BD, 1 MDD).

#### 2.2.2. Imaging protocol & processing

Scanning was performed on a 3 T Siemens PRISMA scanner using a 32-channel head coil and HCP-style sequences (Feinberg et al., 2010; Uğurbil et al., 2013). Specifically, we collected 3D T1-weighted MPRAGE and T2-weighted SPACE acquisitions (0.8 mm^3^) (Glasser et al., 2013) that used navigator-guided scans developed by Tisdall et al. (2012) to compensate for movement. The functional BOLD images were collected with a gradient-echo echo-planar multiband sequence (Feinberg et al., 2010; Uğurbil et al., 2013) using parameters similar to the HCP protocol, i.e., a multiband factor of 6, TR = 800 ms, TE = 30 ms, and 2.4 mm^3^ voxels (72 oblique-axial slices). Two six-minute resting-state runs were collected, each consisting of 460 frames. Data from participants with at least 375 frames retained following motion correction were used in the analyses.

Data was preprocessed using fMRIPrep version 1.3.2 (Esteban et al., 2019). T1-weighted (T1w) images were corrected for intensity non-uniformity (INU) and then skull-stripped. Brain surfaces were reconstructed using Recon-all in FreeSurfer version 6.0.1. Spatial normalization to the ICBM 152 Nonlinear Asymmetrical template version 2009c was performed through nonlinear registration with antsRegistration (ANTs 2.2.0), using brain-extracted versions of both T1w volume and template. Brain tissue segmentation of cerebrospinal fluid (CSF), white-matter (WM), and gray matter (GM) was performed on the brain-extracted T1w using Fast (FSL 5.0.9).

#### 2.2.3. Functional data pre-processing

For each participant, the preprocessing described below was performed for each BOLD run. First, a reference volume and its skull-stripped version were generated using a custom methodology of fMRIPrep. A deformation field was estimated to correct for susceptibility distortions based on two echo-planar imaging (EPI) references with opposing phase-encoding directions, using AFNI’s 3dQwarp. Based on the estimated susceptibility distortion, an unwarped BOLD reference was calculated for a more accurate co-registration with the anatomical reference. The BOLD reference was then co-registered to the T1w reference using bbregister (FreeSurfer 6.0.1) which implements boundary-based registration. Co-registration was configured with nine degrees of freedom to account for distortions remaining in the BOLD reference. Head-motion parameters concerning the BOLD reference (transformation matrices and six corresponding rotation and translation parameters) were estimated before any spatiotemporal filtering using mcflirt (FSL 5.0.9). BOLD runs were slice-time corrected using AFNI 3dTshift. The BOLD time-series were resampled to the fsaverage5 surface. The BOLD time-series (including slice-timing correction) were resampled onto their original native space by applying a single composite transform to correct for head-motion and susceptibility distortions. The BOLD time-series were resampled to MNI152NLin2009cAsym standard space, generating a preprocessed BOLD run in MNI152NLin2009cAsym space.

The following confounding time series were calculated: framewise displacement (FD), DVARS, and three region-wise global signals in Nipype (Gorgolewski et al., 2011). Three global signals were also extracted: cerebral spinal fluid (CSF), white matter (WM), and whole-brain masks. Principal components are estimated after high-pass filtering the preprocessed BOLD time-series (using a discrete cosine filter with 128 s cut-off) for the two CompCor variants: temporal (tCompCor) and anatomical (aCompCor). Six tCompCor components were calculated from the top 5% variable voxels within a mask covering the subcortical regions. This subcortical mask was obtained by heavily eroding the brain mask, which ensured it did not include cortical GM regions. For aCompCor, six components are calculated within the intersection of the aforementioned mask and the union of CSF and WM masks calculated in T1w space, after their projection to the native space of each functional run (using the inverse BOLD-to-T1w transformation). The corresponding confounds file included the head-motion estimates calculated in the correction step. All resamplings were performed with a single interpolation step by composing all the pertinent transformations (i.e., head-motion transform matrices, susceptibility distortion correction when available, and co-registrations to anatomical and template spaces). Gridded (volumetric) resamplings were performed using ‘antsApplyTransforms’ (ANTs), configured with Lanczos interpolation to minimize the smoothing effects of other kernels. Non-gridded (surface) resamplings were performed using mri_vol2surf (FreeSurfer). Filtered frame-wise displacement (FD) measures were used to motion-censor the data, as this approach reduces the influence of motion-related signal changes (Power et al., 2012, 2013, 2014). Time-series were demeaned and detrended. The nuisance waveforms, including motion regressors, whole brain, WM, and CSF, were regressed from the data (Burgess et al., 2016; Ciric et al., 2017). Timepoints were temporally filtered with a bandpass filter (0.01 < f < 0.1).

#### 2.2.4. Individual-specific RSFC

Individual-specific network connectivity metrics were created using an empirically validated template-matching technique (Gordon et al., 2017b). Eight networks were selected a priori and investigated in the analyses: default mode (DMN), cingulo-opercular (CON), dorsal attention (DAN), ventral attention (VAN), salience (SAL), fronto-parietal (FPN), dorsal somato-motor (DSM), and ventral somato-motor (VSM). In brief, this technique identifies each participant’s version of the group-averaged network by matching each vertex’s individual-specific connectivity pattern to a priori Gordon network templates (Gordon et al., 2016). Specifically, pairwise vertex-to-vertex time series correlations were computed, resulting in a 59412 × 59412 matrix for each subject. The top 5% of connections were retained and binarized, creating one spatial map per vertex that indicates vertices with high connectivity to the specific vertex. Dice coefficients were calculated for each vertex, which measure the similarity between the binarized and thresholded spatial map and each a priori network template. Subsequently, each vertex was assigned to the network with the highest associated Dice coefficient. Contiguous vertices were removed if the total surface area was less than 30 mm^2^. Vertices were then parcellated based on network identity and proximity associated with the highest Dice coefficient, resulting in a variable number of parcels per participant.

Average time series for each parcel were extracted, and Pearson correlation matrices were computed between all parcel time series and Fisher *Z*-transformed. Network-level connectivity was calculated by averaging parcel-wise correlations within and between networks, based on each parcel’s assigned network label, to produce a matrix of within and between-network connectivity metrics. Network-level average participation coefficient was used as the between-network connectivity metric. Participation coefficient (PC) is a measure of the diversity in a node’s connections across different networks. It represents the number of connections that a node has outside its network. PCs were calculated at the parcel level using binary graphs thresholded across 1% to 25% tie densities (Marek et al., 2015). To obtain a stable estimate, PCs were averaged across the first 10% of tie densities (Lopez et al., 2020). Finally, the network-level average PCs were calculated by averaging across all parcels assigned to each network, yielding one value per network per participant (Marek et al., 2015). Additionally, the spatial extent of each individual-specific network was measured, defined as the total number of vertices assigned to that network for that individual. In total, eight within-network connectivity metrics, 13 between-network connectivity metrics, eight PC metrics, and eight spatial extent metrics were calculated.

### 2.3. Data analysis

Bayesian hierarchical cumulative logit-link regression models were used to examine the relationships of connectivity (i.e., within-network connectivity and PC) and spatial extent of resting state networks with current and anticipatory MAP. Hierarchical modeling addressed the repeated measurement structure of the EMA data, and models were nested to account for variability between days and participants. A cumulative logit link was employed to accommodate the ordinal nature of the EMA MAP symptom data.

For all analyses, diagnosis was coded as a factor variable with CON as the reference group, resulting in three predictor variables representing the difference between each diagnostic group and community controls. Furthermore, covariates across all models comprised age, gender, and question type—a binary independent variable identifying whether the question was regarding motivation (e.g., “How interested are you in this activity?”) or pleasure (e.g., “How much are you enjoying this activity?”) to account for variability between the concepts.

First, models without connectivity and spatial extent metrics were run to assess diagnostic differences in the variables of interest. They included anticipatory or consummatory MAP as outcome variables and diagnosis and covariates as predictor variables (i.e., age, gender, and question type). These models are later referred to as “diagnostic group models.”

Following these preliminary analyses, models were developed for each combination of outcome (anticipatory or consummatory MAP), predictor (connectivity or spatial extent), and network. Connectivity models included both within-network connectivity and PC as individual predictors. Specifically, each connectivity model included anticipatory or consummatory MAP as the outcome variable and one network’s within-network connectivity and PC as predictor variables. In addition to the covariates previously mentioned (i.e., age, sex, and question type), these models also accounted for movement by including average FD. Furthermore, the models included diagnosis and interactions between diagnosis and the variables of interest to examine transdiagnostic differences. All numerical independent variables were standardized with a mean of 0 and a standard deviation of 1.

The following methods were used to assess the significance of predictor variables: credible intervals (CI), probability of direction (PD), and percent in the region of practical equivalence (ROPE). PD indicates the percentage of posterior predictive samples greater than 0. Percent in ROPE shows the percentage of samples within −0.10 to 0.10, a range intended to represent a practically non-significant effect. Briefly, predictors were considered statistically significant if the odds ratios’ credible intervals did not include 1, and results were discussed. Additionally, if a model resulted in a significant interaction between a predictor of interest and a diagnostic group, a simple slopes analysis was conducted to identify the significance of conditional effects within each group.

#### 2.3.1. Secondary analyses

The network-level PC broadly measures a network’s connectivity with all other networks but does not specify which between-network connectivity metrics are responsible for that relationship. Therefore, if a model showed a significant relationship between a network’s PC and MAP, secondary models were run to identify which between-network connectivity metrics were driving the result. These models were run only within the diagnostic groups that showed a significant relationship between a network’s PC and MAP. Accordingly, secondary models included the MAP variable of interest as the dependent outcome variable, all 13 between-network connectivity metrics as independent variables, and the previously delineated covariates.

## 3. Results

### 3.1. Descriptives

Descriptives are illustrated in Tables 2 and 3. No significant differences were found across groups in age (*F*(3, 140) = 1.76, *p* = .16), parental years of education (*F*(3, 140) = 0.97, *p* = .41), or gender (*χ*^2^(6) = 7.51, *p* = .28). No significant differences were found in motivation and pleasure between the clinical groups with the CAINS (*F*(2,101) = 0.57, *p* = .57). However, significant group differences were found for the MAP-SR (*F*(3, 139) = 8.526, *p* = .00003). Specifically, post-hoc comparisons indicated that CON scored significantly higher than the MDD (mean difference = −11.56, 95% CI [−17.59, −5.53], *p* = .0000106) and SZ (mean difference = −7.41, 95% CI [−13.85, −0.96], *p* = .017) groups.

### 3.2. Diagnostic group models

**Fig. 1.**
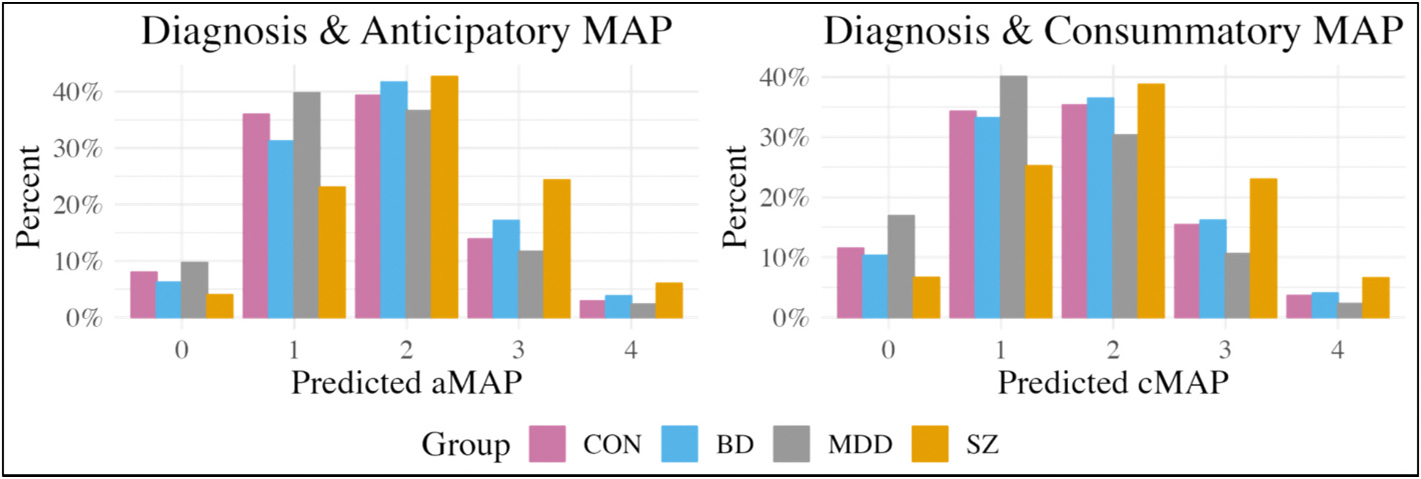
Diagnostic group models. aMAP = anticipatory motivation and pleasure; cMAP = consummatory motivation and pleasure; CON = community controls; BD = bipolar disorder; MDD = major depressive disorder; SZ = schizophrenia/schizoaffective disorder.

As shown in Fig. 1, the results of the diagnostic group models (i.e., the models without connectivity and spatial extent metrics), indicated that SZ were more likely to report greater anticipatory MAP (e^*β*^ = 2.19, 95% CI [1.06, 4.17], PD = 98.28%, 0.86% in ROPE) and consummatory MAP compared to CON (e^*β*^ = 1.97, 95% CI [1.08, 3.75], PD = 98.64%, 0.41% in ROPE). In contrast, BD and MDD showed comparable rates of anticipatory and consummatory MAP compared to CON.

Moreover, these models showed significant main effects of age and question type. These effects indicated that increased age was associated with greater anticipatory and consummatory MAP, and on average, participants rated their anticipatory and consummatory pleasure higher than their motivation.

### 3.3. Connectivity (within-network and participation coefficient) models

**Fig. 2.**
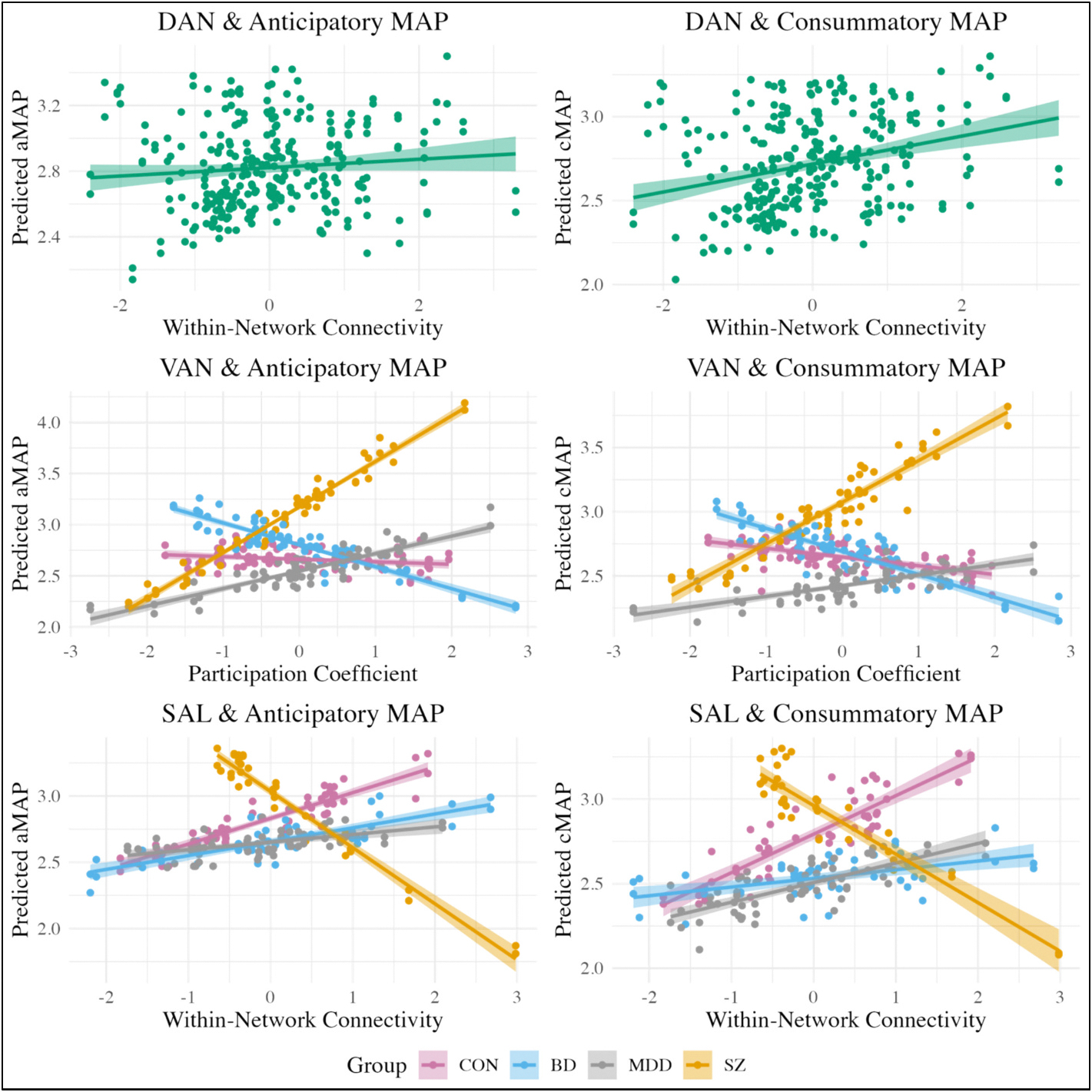
Key significant effects: connectivity & PC models. DAN = dorsal attention; VAN = ventral attention; SAL = salience; CON = community controls; BD = bipolar disorder; MDD = major depressive disorder; SZ = schizophrenia/schizoaffective disorder; PC = participation coefficient.

Key effects of interest for connectivity models are shown in Table 4 and Fig. 2, while full model results are displayed in the Supplementary Material in Tables S1 to S2. Simple slope analyses results are listed in Table 5.

#### 3.3.1. Anticipatory MAP

Regarding the models predicting anticipatory MAP, there was a significant main effect of DAN within-network connectivity, indicating that weaker connectivity within this network was associated with decreased anticipatory MAP across groups. Additionally, two networks showed significant interactions with SZ in predicting anticipatory MAP. As shown in Fig. 2, for SZ specifically, lower PC of the VAN was associated with decreased anticipatory MAP and stronger within-network connectivity of the SAL was linked to decreased anticipatory MAP. The simple slopes analysis further supported these relationships, given that the conditional estimates were statistically significant only in SZ.

#### 3.3.2. Consummatory MAP

The models predicting consummatory MAP exhibited similar patterns (Table 4 and Fig. 2) to those predicting anticipatory MAP. A significant main effect showed that weaker within-network connectivity in the DAN was related to lower consummatory MAP across groups. Furthermore, a significant interaction indicated that decreased VAN PC was associated with lower consummatory MAP for SZ. This conditional estimate was statistically significant for SZ, per the simple-slopes analysis. Another significant interaction demonstrated that, among SZ, stronger within-network connectivity of the SAL was associated with decreased consummatory MAP. However, the simple slopes analysis reflected that the conditional estimate was not significant even within SZ.

#### 3.3.3. Secondary participation coefficient analyses

Secondary analyses aimed to identify the between-network connectivity metrics driving the significant relationships between VAN PC and MAP in SZ. These analyses were limited to SZ because previous findings showed that the relationship between VAN PC and MAP was only significant in this group. Ultimately, the secondary analyses resulted in no effects that reached statistical significance (i.e., the odds ratio credible intervals did not include 1). However, the PD for several between-network connectivity metrics suggested notable effects (i.e., PD > 90). Specifically, these results highlighted the role of connectivity between the VAN and the DMN, FP, and VSM. Notably, connectivity between the VAN and FP was positively associated with both anticipatory and consummatory MAP, whereas connectivity between the VAN and VSM was negatively associated with both outcomes. Additionally, connectivity between the VAN and DMN showed a positive association with anticipatory MAP. The detailed results of these models are provided in Supplementary Tables S5 and S6.

### 3.4. Spatial extent

**Fig. 3.**
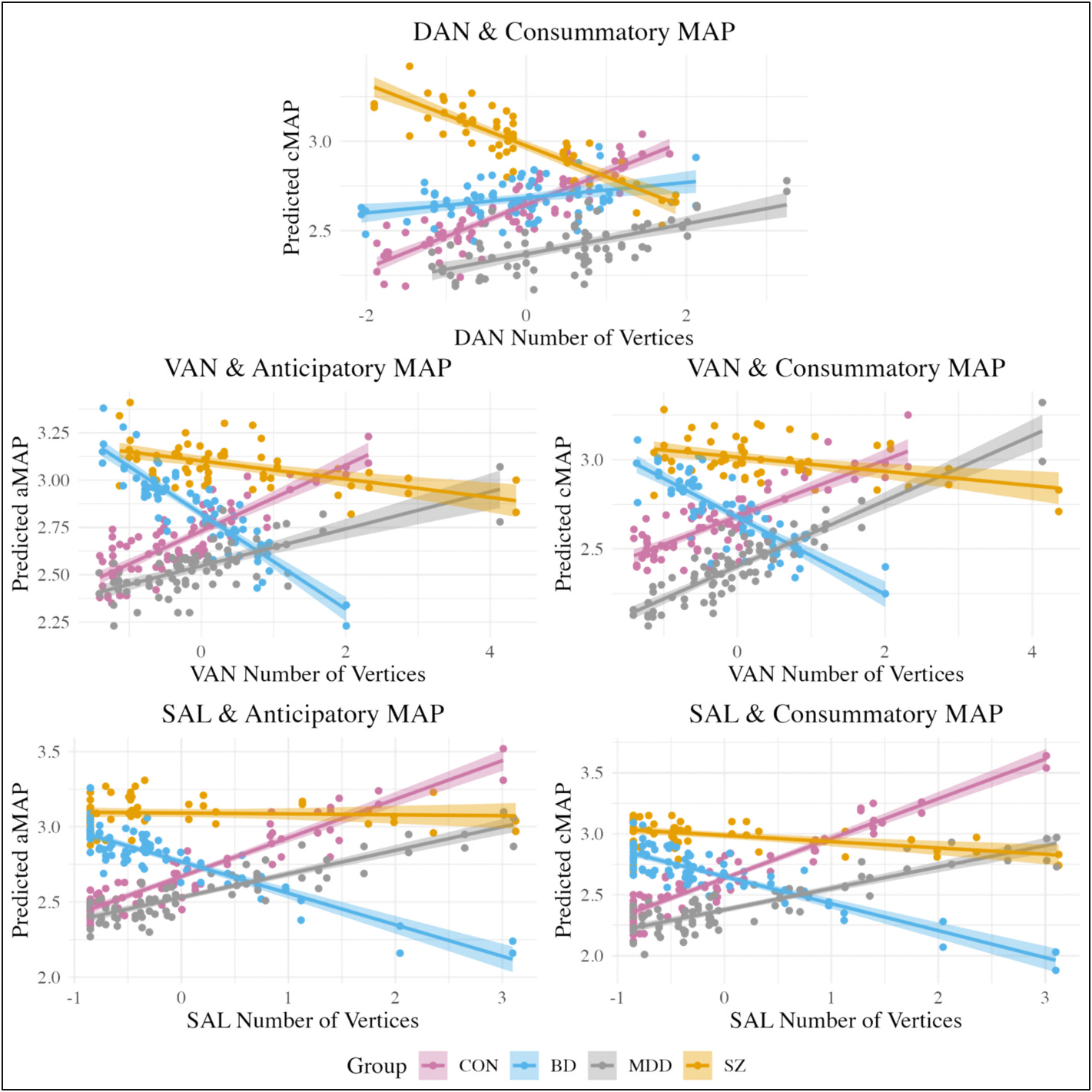
Key significant effects: spatial extent models. DAN = dorsal attention; VAN = ventral attention; SAL = salience; CON = community controls; BD = bipolar disorder; MDD = major depressive disorder; SZ = schizophrenia/schizoaffective disorder.

Key effects of interest for the spatial extent models are shown in Table 4 and Fig. 3. Full model results are available in the Supplementary Material, in Tables S3 to S4. Results of the simple slope analyses are presented in Table 5.

#### 3.4.1. Anticipatory MAP

A significant interaction effect between the number of vertices of the VAN and BD diagnostic group reflected that a larger VAN was associated with lower anticipatory MAP for BD. However, simple slopes analysis revealed that the conditional estimates were not statistically significant in any group.

The model assessing the relationships between anticipatory MAP and the spatial extent of the SAL showed a significant main effect and interaction effect. Specifically, the main effect suggested that increased SAL number of vertices was associated with increased anticipatory MAP across groups. In contrast, the significant interaction effect indicated that a larger SAL was related to decreased anticipatory MAP in BD. Simple slopes analyses showed that none of the conditional estimates were statistically significant.

#### 3.4.2. Consummatory MAP

The models for consummatory MAP align with those for anticipatory MAP, with additional significant effects. First, a significant interaction effect reflected that for SZ, a larger DAN was associated with decreased consummatory MAP. However, simple slopes analysis revealed that the conditional estimates were not statistically significant within any group. In parallel, a significant interaction suggested that increased VAN number of vertices was associated with decreased consummatory MAP for BD. Yet simple slopes analyses demonstrated that the conditional estimates were not significant for any group.

The model assessing the relationship between the spatial extent of the SAL and consummatory MAP revealed a significant main effect and two interactions. These effects indicated that, for CON and MDD, a larger SAL is associated with increased consummatory MAP. In contrast, a greater number of vertices in the SAL was linked to decreased consummatory MAP for BD and SZ, with BD showing a more pronounced decrease. The simple slopes analysis revealed a significant conditional estimate only within CON.

## 4. Discussion

This study investigated whether the connectivity or spatial extent of eight resting-state networks—namely the default mode, cingulo-opercular, dorsal attention, ventral attention, salience, fronto-parietal, dorsal somato-motor, and ventral somato-motor—predicts current or anticipatory motivation and pleasure, as assessed by EMA across individuals with psychotic and mood disorders. Drawing on previous research, we hypothesized that EMA MAP would be related to the connectivity and spatial extent of the default mode, cingulo-opercular, dorsal attention, ventral attention, salience, and fronto-parietal networks. Additionally, we anticipated these relationships would be largely transdiagnostic.

In summary, the diagnostic group analyses demonstrated intact MAP for BD and MDD, while SZ exhibited elevated MAP. We also found a positive relationship between the within-network connectivity of the DAN and MAP, a positive relationship between the PC of the VAN and MAP for SZ, a negative relationship between the within-network connectivity of the SAL and MAP for SZ, and a positive relationship between the spatial extent of the SAL and MAP for CON. Overall, the findings reinforce the complexity of understanding MAP deficits across diagnostic groups and support links between MAP and the connectivity of the DAN, VAN, and SAL. These points will be elaborated on below.

The analyses of the diagnostic group models indicated that SZ reported higher levels of both anticipatory and consummatory MAP, while BD and MDD showed comparable levels to controls. The absence of a deficit in consummatory MAP for individuals with schizophrenia and schizoaffective disorder is unsurprising, as it aligns with previous research showing intact consummatory MAP (Cohen and Minor, 2010; Dowd and Barch, 2012; Kring and Moran, 2008). This prior work suggests that anhedonia may be a misnomer, as individuals with schizophrenia exhibit similar pleasure and hedonic responses to stimuli in the moment and on EMA measures of consummatory pleasure compared to controls (Cohen and Minor, 2010; Dowd and Barch, 2012; Kring and Moran, 2008). However, the lack of anticipatory MAP deficits was unexpected, given the robust literature establishing deficits in anticipatory MAP across assessment modalities (Foussias and Remington, 2010; Galderisi et al., 2018; García-Portilla et al., 2021; Kirkpatrick et al., 2001). Furthermore, the evidence of intact consummatory MAP in the EMA data contrasts with the analysis of the self-report MAP-SR, in which SZ showed reduced MAP compared to CON.

These unexpected findings prompt important questions about how different assessment modalities interact with processes underlying MAP. Previous studies using EMA have found a discrepancy when comparing EMA to retrospective negative symptom assessments in schizophrenia (Gard et al., 2014; Moran et al., 2017a). As demonstrated by Moran et al. (2017a), the discrepancy between EMA and retrospective negative symptom measures is influenced by working memory capacity, such that individuals with more impaired working memory show greater discrepancies. Specifically, retrospective assessments of negative symptoms, such as self-report assessments completed at a single time point, may be limited by working memory impairments seen in psychotic disorders. EMA of these symptoms is theorized to reduce cognitive load, thereby providing a more accurate reflection of their symptomatology. However, future research must continue to deconstruct the discrepancy between retrospective assessments and EMA of negative symptoms and clarify the phenomenology of anhedonia across diagnostic categories before firm assumptions can be made.

Additionally, the absence of MAP deficits in BD and MDD is also surprising and contradicts previous research. Deficits in MAP during behavioral tasks have been demonstrated in individuals with major depressive disorder, with some work noting specific deficits in anticipatory MAP (Sherdell et al., 2012; Treadway et al., 2012). Along with the findings for SZ, the analysis of self-report MAP-SR scores deviated from the EMA results. In this analysis, MDD also showed reduced MAP compared to CON. Overall, previous research indicates deficits in MAP in MDD, which diverges from the EMA findings but align with the results from self-report MAP-SR data. Accordingly, future research should continue exploring why differences in MDD deficits vary depending on the methodology.

While little research has been done on MAP deficits in bipolar disorder, studies have established the presence of negative symptoms (Strauss and Cohen, 2017). Of note, BD showed comparable MAP-SR scores to CON. There is limited research on BD, and a deficit in MAP is not supported by either EMA or MAP-SR data in our study. There are indications in prior literature that the phase of illness may be an important factor, with motivation differences potentially varying between manic and depressed phases (Yang et al., 2021). Within our study, the majority of participants in the BD group were euthymic, which may have contributed to the lack of MAP deficits within this group. In sum, the results suggest that more research is needed to confirm and deconstruct MAP deficits in BD.

The results above focused on group-level differences in EMA and self-report measures of MAP. Additional analyses focused on how individual differences in FC and the spatial extent of resting-state networks relate to MAP across and within diagnostic groups. Therefore, while the group-level differences did not reveal any significant impairments for the clinical groups, the subsequent analyses allow us to interpret individual differences in MAP in relation to variation in functional connectivity and spatial extent, both within and across diagnostic groups. The findings emphasized the importance of three resting-state networks—the DAN, VAN, and SAL—each of which will be discussed in turn.

We found that within-network connectivity of the DAN was positively associated with anticipatory and consummatory MAP across groups. However, the DAN’s PC did not show any significant relationships. Although the spatial extent of the DAN was significantly related to consummatory MAP in SZ, the simple-slopes analysis indicated that this conditional estimate was not significant when examined within SZ. Accordingly, these spatial extent results can be considered preliminary at best and should be followed up with future research before definitive conclusions can be drawn.

The DAN has been demonstrated to play a role in cognitive control by facilitating top-down control of visuospatial stimulus-response selection (Corbetta and Shulman, 2002; Uddin et al., 2019). It may help to orient visuospatial attention based on current goals (Corbetta et al., 2008; Corbetta and Shulman, 2002). Research has previously implicated the DAN in general negative symptoms, not specific to MAP symptoms. Biondi et al. (2024) demonstrated that PANSS lack of spontaneity and flow of conversation was positively associated with the between-network connectivity of the DAN and VAN, and Mellem et al. (2020) highlighted the role of the DAN in general negative symptoms. Nonetheless, many studies have not found any association between connectivity of the DAN and negative symptoms (Fan et al., 2022; Gallucci et al., 2024; Rong et al., 2023; Santo-Angles et al., 2021; Tepper et al., 2023; Wang et al., 2020; Wang et al., 2023; Zhang et al., 2023).

Considering the potential role of the DAN, the observed associations may indicate a greater capacity to direct attention to goal-relevant or motivationally salient information. Therefore, increased connectivity within the DAN might indirectly support MAP by enabling individuals to attend to positive cues and develop a more positive evaluation of current and anticipated pleasure states. Future research could potentially test this interpretation through intervention studies in which individuals with schizophrenia are prompted to orient their attention to salient cues that might bolster their motivation and pleasure.

Regarding the VAN, we observed a positive relationship between PC and both anticipatory and consummatory MAP, but only for SZ. However, secondary analyses aimed at identifying which specific between-network connectivity metrics drove this result showed that no between-network connectivity metrics were significantly related to MAP within SZ, suggesting a more diffuse relationship. Additionally, no significant relationships were found between MAP and the VAN’s within-network connectivity. While the spatial extent of the VAN showed a significant relationship with anticipatory and consummatory MAP in BD, the simple-slopes analysis indicated that these conditional estimates were not statistically significant. Given the conflicting results, these findings on spatial extent are considered preliminary and require further investigation in future research.

The VAN has been theorized to facilitate exogenous salience detection, namely guiding attention towards behaviorally relevant external stimuli, especially unattended or low-frequency stimuli (Corbetta and Shulman, 2002). This network is thought to guide visuospatial attention and function as a sort of “circuit breaker” for the DAN (Corbetta et al., 2008; Corbetta and Shulman, 2002). Previous studies have found associations between the VAN and negative symptoms broadly not specifically MAP symptoms (Biondi et al., 2024; Mellem et al., 2020), though the specific directionality of these relationships is unclear.

When integrating the lack of significant VAN between-network relationships in the secondary analyses with the primary analyses, our results suggest that a broader pattern of decreased integration between the VAN and other neural networks is associated with decreased MAP in individuals with schizophrenia or schizoaffective disorder, rather than specific communication between the VAN and any one neural network. At the same time, the absence of VAN between-network effects tempers this interpretation, indicating that any such associations may be modest. Nonetheless, these findings might imply that disruptions in the ability to capture salient information outside of the individual’s current attention focus and integrate it with other processing and representational streams could lead to lower MAP ratings. As discussed above, this hypothesis could be explored by examining interventions that orient individuals with schizophrenia to salient cues in their environment, which could potentially drive goal-directed behavior.

Lastly, we observed a negative relationship between the SAL’s within-network connectivity and anticipatory MAP, but only among SZ. No significant relationship was found between the SAL’s PC and MAP. Additionally, the spatial extent analyses found a positive relationship between the number of vertices in the SAL and consummatory MAP, but simple slopes analysis revealed this was only significant within CON. The SAL’s role is believed to be to broadly detect and identify internal and external behaviorally relevant environmental stimuli. The literature has demonstrated that this network plays an important role in cognitive control, social, affective, and attentional processes (Uddin, 2015; Uddin et al., 2019). Previous research that has investigated FC and schizophrenia more broadly has demonstrated a pattern of reduced connectivity in individuals with schizophrenia, with emphasis on the DMN and SAL (Dong et al., 2018; Fornito et al., 2012; Li et al., 2019; O’Neill et al., 2019; Pettersson-Yeo et al., 2011b).

While not specific to the domain of MAP negative symptoms, several studies have demonstrated an association between general negative symptoms and FC of the SAL in schizophrenia. Namely, Lee et al. (2018) demonstrated a negative relationship between negative symptoms and within-network connectivity of the SAL. Additionally, Wang et al. (2020) found that IS-FC metrics were associated with connectivity within and between the SAL. This finding was not found when they utilized a traditional group-averaged atlas-based approach. However, while these and other studies implicated the role of the SAL (Kebets et al., 2019; Mellem et al., 2020), several found no relationships between negative symptoms and the SAL for individuals with schizophrenia (Mamah et al., 2013; Moser et al., 2018; Rong et al., 2023).

Overall, given the function of the SAL, the results might suggest that a disruption in how salient information is processed contributes to lower MAP within individuals with schizophrenia/schizoaffective disorder. Thus, those with impaired ability to detect salient stimuli might fail to pick up on and attend to cues that drive pleasure and motivation. Additionally, the spatial extent analyses resulted in a positive relationship specific to CON. This might suggest dysfunction of the SAL in clinical groups, in that the relationship between SAL size and MAP becomes uncoupled. Notably, the direction of this relationship differs from the observed negative relationship between SAL connectivity and MAP in the schizophrenia/schizoaffective group. Since this finding is specific to spatial extent, it highlights significant differences in these metrics and possibly what they represent in terms of network function.

Globally, the findings emphasize the role of brain networks that support attentional processes in MAP. Our initial hypotheses anticipated mostly transdiagnostic relationships. The findings regarding DAN connectivity largely cut across diagnoses and support our expectations. In contrast, the relationships involving the connectivity of the VAN and SAL were specific to schizophrenia/schizoaffective disorder. These results reflect that the neural processes underlying MAP vary depending on diagnosis. Specifically, the findings suggest that VAN and SAL connectivity patterns related to MAP differ between individuals diagnosed with psychotic or mood disorders.

The previous literature, although largely conflicting, also suggested potential roles of the DMN, CO, and FP. Accordingly, we initially hypothesized that MAP symptoms would be significantly associated with the connectivity and spatial extent of these networks. However, we found no significant relationships with any of the three networks. This discrepancy might stem from methodological differences. Previous studies generally focused on global negative symptoms, assessed through clinician-rated or self-report measures at a single time point, and used group-averaged FC. Conversely, our approach involved EMA and IS-FC, which may account for the differing results.

There are several limitations to consider in this study. First, EMA MAP reflects an individual’s subjective evaluation of their motivation and pleasure states; however, it does not account for engagement in goal-directed activities, which is a crucial factor when examining MAP impairments. Second, the EMA MAP assessment method used in the study does not differentiate between primary and secondary negative symptoms. Third, the literature has consistently shown that longer scanning times produce more reliable functional connectivity data (Birn et al., 2013). Gordon et al. (2017b) demonstrated that reliability for resting-state scans plateaus at around 20 min of cleaned data. This is especially relevant for individuals with psychotic disorders, who tend to generate more motion-related noise on average (Makowski et al., 2019). However, in this study, participants had an average of approximately 10 min of cleaned data. Fourth, the sample sizes for each group were not large, and thus some null findings may reflect low power. Fifth, the study employed a template matching approach, which is not fully individualized and therefore masks some inter-individual variability in functional connectivity.

Overall, the results support connections between MAP and the connectivity and spatial extent of the DAN, VAN, and SAL. While some effects extend across groups, several relationships were specific to individuals with schizophrenia or schizoaffective disorder. This highlights the role of attentional processes in MAP and suggests that neural mechanisms underlying these functions vary depending on diagnosis. Additionally, the findings emphasize the complex nature of MAP deficits in psychotic and mood disorders and underscore the need for future research to consider inter-individual variability to fully understand the phenomenology and neural basis of these deficits.

## Supplementary Material

1

## Figures and Tables

**Table 1:** EMA MAP-negative symptoms questions and possible answers.

Construct	Question	Answers

Current MAP	What are you doing right now?	“Eating/drinking”, “TV/radio/reading/computer”, “Work/school”, “Socializing”, “Exercising”, “Running an errand”, “Smoking”, “Cleaning/hygiene/chores”, “Doctor/therapist”, “Entertainment away from home”, “Nothing in particular”, “Sleeping”
	How much are you enjoying this activity?	“Not at all”, “A little bit”, “Moderately”, “Quite a bit”, “Extremely”
	How interested are you in this activity?	“Not at all”, “A little bit”, “Moderately”, “Quite a bit”, “Extremely”
Anticipatory MAP	In the next 2 to 3 h, which of these activities do you think you are most likely to do?	“Eating/drinking”, “TV/radio/reading/computer”, “Work/school”, “Socializing”, “Exercising”, “Running an errand”, “Smoking”, “Cleaning/hygiene/chores”, “Doctor/therapist”, “Entertainment away from home”, “Nothing in particular”, “Sleeping”
	How much do you think you will enjoy this activity?	“Not at all”, “A little bit”, “Moderately”, “Quite a bit”, “Extremely”
	How interested do you think you will be in this activity?	“Not at all”, “A little bit”, “Moderately”, “Quite a bit”, “Extremely”

**Table 2:** Sample demographics.

Category	N (%)

Diagnosis/gender	
Controls (CON)	40 (28%)
Female/non-binary	21 (52%)
Bipolar disorder (BD)	38 (26%)
Female/non-binary	25 (66%)
Major depressive disorder (MDD)	37 (26%)
Female/non-binary	23 (62%)
Schizophrenia/schizoaffective (SZ)	29 (20%)
Female/non-binary	12 (41%)
Race	
White	77 (53.5%)
Black	55 (38.2%)
White & Black	4 (2.8%)
Filipino	2 (1.4%)
Other race	2 (1.4%)
Japanese	1 (0.7%)
Native American	1 (0.7%)
Other Asian	1 (0.7%)
White & Native American	1 (0.7%)
Ethnicity	
Not Hispanic/Latino	139 (96.5%)
Hispanic/Latino	5 (3.5%)

**Table 3:** Sample characteristics.

Variable	CON			BD			MDD			SZ			Statistics	
	Mean	Median	SD	Mean	Median	SD	Mean	Median	SD	Mean	Median	SD	F	Post-hoc

Age	35.15	35.00	7.62	36.43	39.00	8.03	32.65	32.00	8.21	34.42	35.00	8.32	*F*(3, 140) = 1.76 *p* = .16	-
WTAR FSIQ	105.59	108.00	9.30	107.99	112.00	10.52	104.28	108.00	11.51	96.68	98.00	13.38	F(3, 139) = 6.26 ***p* < .001**	SZ < CON & BD & MDD
BPRS negative	-	-	-	5.93	5.00	2.53	6.22	6.00	1.97	8.03	8.00	2.47	*F*(2, 101) = 7.87 ***p* < .001**	SZ > BD & MDD
BPRS positive	-	-	-	4.02	3.00	2.02	3.42	3.00	0.99	9.75	10.00	4.34	F(2,101) = 55.87 ***p* < .001**	SZ > BD & MDD
BPRS disorganization	-	-	-	4.81	4.00	1.98	4.23	4.00	0.60	6.35	6.00	2.26	F(2,101) = 14.75 ***p* < .001**	SZ > BD & MDD
BPRS mania	-	-	-	7.31	5.00	3.77	5.34	5.00	0.61	7.29	7.00	2.64	F(2,101) = 6.72 ***p* = .002**	MDD < SZ & BD
BPRS depression	-	-	-	10.36	9.00	4.62	13.30	13.00	3.42	9.68	8.00	4.22	F(2,101) = 7.66 ***p* < .001**	MDD > BD & SZ
CAINS MAP	-	-	-	12.85	13.00	6.09	13.68	14.00	5.64	13.89	13.00	7.35	*F*(2, 101) = 0.57 *p* = .57	-
CAINS EXP	-	-	-	0.98	0.00	2.31	1.38	0.00	1.98	2.75	2.00	2.86	F(2, 101) = 5.07 ***p* = .008**	SZ > BD
CESD10	5.38	6.00	3.38	11.65	11.00	6.20	15.01	15.00	4.96	10.51	11.00	6.80	F(3, 140) = 21.05 ***p* < .001**	CON < SZ & BD < MDD
MAP-SR	42.04	43.00	9.50	35.73	36.00	9.09	31.09	31.00	9.35	34.55	33.00	12.51	F(3, 139) = 8.53 ***p* < .001**	CON > MDD & SZ

CON = community controls; BD = bipolar disorder; MDD = major depressive disorder; SZ = schizophrenia/schizoaffective disorder.

Bold text indicates statistical significance (i.e., p < .05).

**Table 4:** Key significant effects: connectivity and PC models & spatial extent models.

Outcome	Predictor		℮^*β*^	95% CI	PD	ROPE
	Network	Variable				

Connectivity and PC models						
aMAP	DAN	Within-network	1.73	[1.03, 2.85]	98.19%	1.95%
aMAP	VAN	Interaction: PC & SZ	2.72	[1.31, 5.61]	99.62%	0%
aMAP	SAL	Interaction: within-network & SZ	0.28	[0.11, 0.70]	99.66%	0%
cMAP	DAN	Within-network	1.60	[1.05, 2.43]	98.54%	1.85%
cMAP	VAN	Interaction: PC & SZ	2.00	[1.07, 3.72]	98.51%	0.57%
cMAP	SAL	Interaction: within-network & SZ	0.37	[0.17, 0.80]	99.31%	0%
Spatial extent models						
aMAP	VAN	Interaction: number of vertices & BD	0.44	[0.19, 0.98]	97.86%	1.59%
aMAP	SAL	Number of vertices	1.66	[1.00, 2.71]	97.41%	3.03%
		Interaction: number of vertices & BD	0.38	[0.18, 0.80]	99.48%	0%
cMAP	DAN	Interaction: number of vertices & SZ	0.51	[0.27, 0.96]	98.13%	1.32%
cMAP	VAN	Interaction: number of vertices & BD	0.50	[0.25, 0.98]	97.72%	1.80%
cMAP	SAL	Number of vertices	1.79	[1.17, 2.75]	99.54%	0%
		Interaction: number of vertices & BD	0.37	[0.20, 0.68]	99.92%	0%
		Interaction: number of vertices & SZ	0.53	[0.29, 0.95]	98.28%	1.36%

aMAP = anticipatory motivation and pleasure; cMAP = consummatory motivation and pleasure; DAN = dorsal attention; VAN = ventral attention; SAL = salience; PC = participation coefficient; SZ = schizophrenia/schizoaffective disorder; BD = bipolar disorder.

**Table 5:** Simple slopes analysis: conditional effect estimates.

Outcome	Predictor		℮^*β*^ [95% CI]			
	Network	Variable	CON	BD	MDD	SZ

Connectivity and PC models						
aMAP	VAN	PC	0.94 [0.60, 1.48]	0.66 [0.38, 1.12]	1.51 [0.92, 2.50]	**2.54 [1.41, 4.63]**
aMAP	SAL	Within-network	1.46 [0.84, 2.45]	1.31 [0.75, 2.24]	1.11 [0.62, 1.97]	**0.41 [0.20, 0.85]**
cMAP	VAN	PC	0.92 [0.61, 1.35]	0.69 [0.44, 1.08]	1.20 [0.78, 1.84]	**1.84 [1.12, 3.03]**
cMAP	SAL	Within-network	1.53 [0.94, 2.46]	1.16 [0.72, 1.86]	1.24 [0.75, 2.08]	0.56 [0.30, 1.06]
Spatial extent models						
aMAP	VAN	Number of vertices	1.46 [0.86, 2.47]	0.64 [0.33, 1.23]	1.17 [0.70, 1.94]	0.92 [0.58, 1.47]
aMAP	SAL	Number of vertices	1.66 [1.00, 2.72]	0.64 [0.35, 1.16]	1.44 [0.89, 2.27]	1.01 [0.60, 1.67]
cMAP	DAN	Number of vertices	1.40 [0.92, 2.11]	1.03 [0.64, 1.64]	1.22 [0.79, 1.81]	0.71 [0.42, 1.19]
cMAP	VAN	Number of vertices	1.35 [0.89, 2.06]	0.67 [0.39, 1.17]	1.48 [0.95, 2.25]	0.93 [0.65, 1.39]
cMAP	SAL	Number of vertices	**1.79 [1.18, 2.74]**	0.65 [0.41, 1.06]	1.42 [0.97, 2.02]	0.94 [0.62, 1.44]

aMAP = anticipatory motivation and pleasure; cMAP = consummatory motivation and pleasure; VAN = ventral attention; SAL = salience; DAN = dorsal attention; PC = participation coefficient; CON = community controls; BD = bipolar disorder; MDD = major depressive disorder; SZ = schizophrenia/schizoaffective disorder. Bold text indicates statistical significance (i.e., 95% CI does not include 1).

## Appendix A. Supplementary data

Supplementary data to this article can be found online at https://doi.org/10.1016/j.schres.2026.02.016.
