# Supplementary material for: Negative symptoms and resting state functional connectivity: Leveraging ecological momentary assessment and individual-specific techniques
Source: Schizophr Res. Author manuscript; Available in PMC 2026 Jun 2. (PMC13228094; doi:10.1016/j.schres.2026.02.016)
Supplement: 1 [file NIHMS2168190-supplement-1.docx]

**Supplementary Material**

**Negative Symptoms and Resting State Functional Connectivity: Leveraging Ecological Momentary Assessment and Individual-Specific Techniques**

Nada Dalloul^a^, Sridhar Kandala^b^, Erin Moran, Ph.D.^a^, Deanna M. Barch, Ph.D.^a,c,d^

^a^ Department of Psychological & Brain Sciences, Washington University, St. Louis, MO

^b^ Laboratory of Behavioral Neuroscience, National Institute on Aging, National Institutes of Health, Baltimore, MD

^c^ Department of Psychiatry, Washington University School of Medicine, St. Louis, MO

^d^ Department of Radiology, Mallinckrodt Institute of Radiology, Washington University School of Medicine, St. Louis, MO

Corresponding author: Nada Dalloul

Washington University

Department of Psychological & Brain Sciences

Box 1125

One Brookings Drive

St. Louis, Mo. 63130

Phone: 314-935-8547

Fax: 314-935-8790

Email: d.nada@wustl.edu

Supplemental Table 1. aMAP Connectivity Models

| **Predictor** | **DMN** | **FP** | **DA** | **VA** | **Sal** | **CO** | **DSM** | **VSM** |
| --- | --- | --- | --- | --- | --- | --- | --- | --- |
| **PC** | ℮𝛽 = 1.18 95% CI[0.66, 2.11] PD = 71.33% ROPE = 23.73% | ℮𝛽 = 1.05 95% CI[0.59, 1.85] PD = 56.96% ROPE = 28.93% | ℮𝛽 = 0.97 95% CI[0.61, 1.57] PD = 54.5% ROPE = 33.79% | ℮𝛽 = 0.94 95% CI[0.6, 1.48] PD = 61.45% ROPE = 33.62% | ℮𝛽 = 1.1 95% CI[0.58, 2.1] PD = 61.64% ROPE = 23.8% | ℮𝛽 = 1.05 95% CI[0.64, 1.73] PD = 58.26% ROPE = 31.32% | ℮𝛽 = 1.15 95% CI[0.71, 1.86] PD = 70.89% ROPE = 29.27% | ℮𝛽 = 1.24 95% CI[0.77, 2.01] PD = 81.98% ROPE = 23.2% |
| **WN FZ Cor** | ℮𝛽 = 1.11 95% CI[0.65, 1.9] PD = 65.23% ROPE = 27.73% | ℮𝛽 = 1.28 95% CI[0.75, 2.16] PD = 81.76% ROPE = 20.03% | ℮𝛽 = 1.73 95% CI[1.03, 2.85] PD = 98.19% ROPE = 1.95% | ℮𝛽 = 0.99 95% CI[0.61, 1.61] PD = 51.32% ROPE = 32.87% | ℮𝛽 = 1.45 95% CI[0.84, 2.49] PD = 90.99% ROPE = 12.52% | ℮𝛽 = 1.11 95% CI[0.68, 1.8] PD = 66.28% ROPE = 29.82% | ℮𝛽 = 1.13 95% CI[0.72, 1.77] PD = 71.26% ROPE = 30.93% | ℮𝛽 = 0.86 95% CI[0.6, 1.23] PD = 80.26% ROPE = 31.33% |
| **Age** | ℮𝛽 = 1.03 95% CI[1, 1.07] PD = 97.42% ROPE = 100% | ℮𝛽 = 1.03 95% CI[1, 1.07] PD = 96.68% ROPE = 100% | ℮𝛽 = 1.04 95% CI[1.01, 1.07] PD = 98.78% ROPE = 100% | ℮𝛽 = 1.04 95% CI[1.01, 1.08] PD = 99.23% ROPE = 100% | ℮𝛽 = 1.02 95% CI[0.98, 1.06] PD = 87.91% ROPE = 100% | ℮𝛽 = 1.03 95% CI[1, 1.07] PD = 97.47% ROPE = 100% | ℮𝛽 = 1.03 95% CI[1, 1.07] PD = 97.7% ROPE = 100% | ℮𝛽 = 1.03 95% CI[0.99, 1.06] PD = 95.24% ROPE = 100% |
| **Gender** | ℮𝛽 = 0.68 95% CI[0.41, 1.15] PD = 92.39% ROPE = 11.61% | ℮𝛽 = 0.74 95% CI[0.44, 1.26] PD = 86.12% ROPE = 17.09% | ℮𝛽 = 0.75 95% CI[0.45, 1.25] PD = 86.69% ROPE = 17.55% | ℮𝛽 = 0.69 95% CI[0.41, 1.14] PD = 92.54% ROPE = 11.99% | ℮𝛽 = 0.79 95% CI[0.46, 1.37] PD = 79.69% ROPE = 20.72% | ℮𝛽 = 0.7 95% CI[0.41, 1.2] PD = 90.94% ROPE = 13.28% | ℮𝛽 = 0.65 95% CI[0.39, 1.09] PD = 94.97% ROPE = 8.11% | ℮𝛽 = 0.74 95% CI[0.44, 1.25] PD = 87.94% ROPE = 16.12% |
| **GroupBD** | ℮𝛽 = 1.18 95% CI[0.59, 2.34] PD = 67.91% ROPE = 21.28% | ℮𝛽 = 1.28 95% CI[0.64, 2.55] PD = 75.19% ROPE = 18.22% | ℮𝛽 = 1.34 95% CI[0.68, 2.61] PD = 80.14% ROPE = 16.65% | ℮𝛽 = 1.31 95% CI[0.68, 2.53] PD = 79.41% ROPE = 18.12% | ℮𝛽 = 0.67 95% CI[0.32, 1.39] PD = 85.7% ROPE = 12.85% | ℮𝛽 = 1.26 95% CI[0.63, 2.48] PD = 74.57% ROPE = 19.38% | ℮𝛽 = 1.36 95% CI[0.68, 2.73] PD = 80.84% ROPE = 15.58% | ℮𝛽 = 1.21 95% CI[0.6, 2.41] PD = 70.76% ROPE = 20.34% |
| **GroupMDD** | ℮𝛽 = 0.68 95% CI[0.34, 1.42] PD = 85.22% ROPE = 12.82% | ℮𝛽 = 0.77 95% CI[0.37, 1.59] PD = 76.41% ROPE = 17.32% | ℮𝛽 = 0.76 95% CI[0.38, 1.51] PD = 78.59% ROPE = 17.49% | ℮𝛽 = 0.75 95% CI[0.38, 1.47] PD = 80.04% ROPE = 17.24% | ℮𝛽 = 0.73 95% CI[0.36, 1.47] PD = 81.59% ROPE = 15.64% | ℮𝛽 = 0.7 95% CI[0.34, 1.43] PD = 84.41% ROPE = 13.88% | ℮𝛽 = 0.63 95% CI[0.31, 1.27] PD = 90.19% ROPE = 10.17% | ℮𝛽 = 0.74 95% CI[0.37, 1.5] PD = 80.26% ROPE = 15.99% |
| **GroupSZ** | ℮𝛽 = 2.19 95% CI[1.03, 4.63] PD = 97.84% ROPE = 1.29% | ℮𝛽 = 2.43 95% CI[1.11, 5.38] PD = 98.77% ROPE = 0% | ℮𝛽 = 2.57 95% CI[1.2, 5.53] PD = 99.36% ROPE = 0% | ℮𝛽 = 2.7 95% CI[1.34, 5.48] PD = 99.66% ROPE = 0% | ℮𝛽 = 1.5 95% CI[0.66, 3.4] PD = 83.23% ROPE = 12.64% | ℮𝛽 = 2.3 95% CI[1.06, 4.99] PD = 98.24% ROPE = 0.75% | ℮𝛽 = 2.28 95% CI[1.04, 4.98] PD = 98.04% ROPE = 1.03% | ℮𝛽 = 2.28 95% CI[1.05, 4.86] PD = 98.21% ROPE = 0.77% |
| **FD** | ℮𝛽 = 0.82 95% CI[0.62, 1.08] PD = 91.92% ROPE = 22.72% | ℮𝛽 = 0.9 95% CI[0.68, 1.19] PD = 77.99% ROPE = 43.58% | ℮𝛽 = 0.94 95% CI[0.72, 1.23] PD = 68.36% ROPE = 51.05% | ℮𝛽 = 0.84 95% CI[0.65, 1.11] PD = 89.28% ROPE = 29.66% | ℮𝛽 = 1.02 95% CI[0.77, 1.35] PD = 56.28% ROPE = 54.06% | ℮𝛽 = 0.91 95% CI[0.69, 1.2] PD = 74.76% ROPE = 45.38% | ℮𝛽 = 0.96 95% CI[0.73, 1.25] PD = 63.16% ROPE = 53.74% | ℮𝛽 = 0.93 95% CI[0.71, 1.23] PD = 68.65% ROPE = 49.45% |
| **Measure** | ℮𝛽 = 1.06 95% CI[1.02, 1.11] PD = 99.79% ROPE = 98.88% | ℮𝛽 = 1.06 95% CI[1.02, 1.11] PD = 99.79% ROPE = 99.03% | ℮𝛽 = 1.06 95% CI[1.02, 1.11] PD = 99.81% ROPE = 98.67% | ℮𝛽 = 1.06 95% CI[1.02, 1.11] PD = 99.67% ROPE = 98.67% | ℮𝛽 = 1.05 95% CI[1, 1.1] PD = 96.48% ROPE = 100% | ℮𝛽 = 1.06 95% CI[1.02, 1.11] PD = 99.76% ROPE = 98.82% | ℮𝛽 = 1.06 95% CI[1.02, 1.11] PD = 99.74% ROPE = 98.69% | ℮𝛽 = 1.06 95% CI[1.02, 1.11] PD = 99.77% ROPE = 98.75% |
| **PC x Group BD** | ℮𝛽 = 0.53 95% CI[0.23, 1.24] PD = 93.23% ROPE = 5.89% | ℮𝛽 = 0.77 95% CI[0.36, 1.64] PD = 75.91% ROPE = 17.27% | ℮𝛽 = 0.84 95% CI[0.4, 1.76] PD = 67.66% ROPE = 19.64% | ℮𝛽 = 0.7 95% CI[0.34, 1.39] PD = 84.31% ROPE = 14.61% | ℮𝛽 = 0.71 95% CI[0.26, 1.97] PD = 74.78% ROPE = 13.09% | ℮𝛽 = 0.96 95% CI[0.42, 2.18] PD = 53.69% ROPE = 20.55% | ℮𝛽 = 0.68 95% CI[0.31, 1.49] PD = 83.19% ROPE = 13.49% | ℮𝛽 = 0.89 95% CI[0.44, 1.84] PD = 63.19% ROPE = 22.18% |
| **PC x Group MDD** | ℮𝛽 = 1.05 95% CI[0.48, 2.31] PD = 54.95% ROPE = 20.86% | ℮𝛽 = 1.2 95% CI[0.5, 2.89] PD = 65.7% ROPE = 16.91% | ℮𝛽 = 1.42 95% CI[0.73, 2.77] PD = 84.96% ROPE = 14.56% | ℮𝛽 = 1.61 95% CI[0.83, 3.13] PD = 92.12% ROPE = 9.47% | ℮𝛽 = 0.84 95% CI[0.36, 1.96] PD = 65.36% ROPE = 17.25% | ℮𝛽 = 1.24 95% CI[0.6, 2.57] PD = 72.24% ROPE = 19.37% | ℮𝛽 = 1.59 95% CI[0.79, 3.18] PD = 90.15% ROPE = 10.16% | ℮𝛽 = 1.12 95% CI[0.57, 2.21] PD = 63.32% ROPE = 22.98% |
| **PC x Group SZ** | ℮𝛽 = 1.1 95% CI[0.45, 2.71] PD = 58.01% ROPE = 17.94% | ℮𝛽 = 1.09 95% CI[0.48, 2.54] PD = 58.25% ROPE = 19.6% | ℮𝛽 = 1.28 95% CI[0.6, 2.69] PD = 74.24% ROPE = 17.85% | ℮𝛽 = 2.72 95% CI[1.31, 5.61] PD = 99.62% ROPE = 0% | ℮𝛽 = 0.99 95% CI[0.34, 2.96] PD = 50.55% ROPE = 15.15% | ℮𝛽 = 1.39 95% CI[0.72, 2.7] PD = 83.58% ROPE = 15.4% | ℮𝛽 = 0.86 95% CI[0.42, 1.74] PD = 67.34% ROPE = 21.75% | ℮𝛽 = 1.14 95% CI[0.52, 2.47] PD = 63.31% ROPE = 19.88% |
| **WN FZ Cor x Group BD** | ℮𝛽 = 0.5 95% CI[0.21, 1.16] PD = 94.73% ROPE = 5.12% | ℮𝛽 = 0.54 95% CI[0.24, 1.21] PD = 93.47% ROPE = 6.38% | ℮𝛽 = 0.5 95% CI[0.25, 1.02] PD = 97.17% ROPE = 2.78% | ℮𝛽 = 0.66 95% CI[0.32, 1.35] PD = 87.01% ROPE = 12.13% | ℮𝛽 = 0.89 95% CI[0.41, 1.92] PD = 62.13% ROPE = 20.45% | ℮𝛽 = 0.79 95% CI[0.41, 1.57] PD = 74.75% ROPE = 18.87% | ℮𝛽 = 0.91 95% CI[0.48, 1.75] PD = 61.56% ROPE = 24.07% | ℮𝛽 = 0.97 95% CI[0.5, 1.89] PD = 53.33% ROPE = 24.64% |
| **WN FZ Cor x Group MDD** | ℮𝛽 = 1.12 95% CI[0.54, 2.33] PD = 62.28% ROPE = 20.99% | ℮𝛽 = 0.92 95% CI[0.42, 2] PD = 58.8% ROPE = 20.37% | ℮𝛽 = 0.53 95% CI[0.26, 1.08] PD = 95.81% ROPE = 4.78% | ℮𝛽 = 1.06 95% CI[0.55, 2.03] PD = 57.54% ROPE = 25.03% | ℮𝛽 = 0.76 95% CI[0.35, 1.67] PD = 74.39% ROPE = 16.19% | ℮𝛽 = 0.95 95% CI[0.45, 1.99] PD = 55.53% ROPE = 22.16% | ℮𝛽 = 1.37 95% CI[0.69, 2.73] PD = 81.67% ROPE = 16.22% | ℮𝛽 = 1.37 95% CI[0.66, 2.87] PD = 80.62% ROPE = 15.86% |
| **WN FZ Cor x Group SZ** | ℮𝛽 = 0.67 95% CI[0.27, 1.67] PD = 80.96% ROPE = 12.74% | ℮𝛽 = 0.74 95% CI[0.32, 1.73] PD = 75.96% ROPE = 14.76% | ℮𝛽 = 0.54 95% CI[0.27, 1.12] PD = 95.2% ROPE = 5.74% | ℮𝛽 = 1.43 95% CI[0.65, 3.17] PD = 81.21% ROPE = 14.17% | ℮𝛽 = 0.28 95% CI[0.11, 0.7] PD = 99.66% ROPE = 0% | ℮𝛽 = 0.65 95% CI[0.31, 1.35] PD = 87.66% ROPE = 11.05% | ℮𝛽 = 0.94 95% CI[0.41, 2.13] PD = 56.04% ROPE = 20.07% | ℮𝛽 = 0.62 95% CI[0.24, 1.62] PD = 83.8% ROPE = 10.38% |

Supplemental Table 2. cMAP Connectivity Models

| **Predictor** | **DMN** | **FP** | **DA** | **VA** | **Sal** | **CO** | **DSM** | **VSM** |
| --- | --- | --- | --- | --- | --- | --- | --- | --- |
| **PC** | ℮𝛽 = 1.12 95% CI[0.68, 1.84] PD = 67.42% ROPE = 28.91% | ℮𝛽 = 1.02 95% CI[0.62, 1.66] PD = 53.05% ROPE = 33.08% | ℮𝛽 = 0.9 95% CI[0.61, 1.34] PD = 69.93% ROPE = 35.45% | ℮𝛽 = 0.91 95% CI[0.62, 1.35] PD = 67.42% ROPE = 37.12% | ℮𝛽 = 0.96 95% CI[0.55, 1.68] PD = 55.42% ROPE = 29.11% | ℮𝛽 = 1.05 95% CI[0.69, 1.59] PD = 58.87% ROPE = 37.45% | ℮𝛽 = 1.2 95% CI[0.79, 1.83] PD = 81.09% ROPE = 26.31% | ℮𝛽 = 1.38 95% CI[0.92, 2.07] PD = 94.23% ROPE = 11.98% |
| **WN FZ Cor** | ℮𝛽 = 1.02 95% CI[0.64, 1.63] PD = 52.69% ROPE = 34.26% | ℮𝛽 = 1.16 95% CI[0.74, 1.83] PD = 74.46% ROPE = 28.47% | ℮𝛽 = 1.6 95% CI[1.05, 2.43] PD = 98.54% ROPE = 1.85% | ℮𝛽 = 1 95% CI[0.65, 1.54] PD = 50.45% ROPE = 38.45% | ℮𝛽 = 1.53 95% CI[0.96, 2.45] PD = 96.28% ROPE = 6.46% | ℮𝛽 = 1.14 95% CI[0.75, 1.72] PD = 72.93% ROPE = 31.91% | ℮𝛽 = 1.24 95% CI[0.84, 1.83] PD = 85.75% ROPE = 24.24% | ℮𝛽 = 0.96 95% CI[0.71, 1.3] PD = 61.31% ROPE = 49.37% |
| **Age** | ℮𝛽 = 1.03 95% CI[1, 1.06] PD = 97.88% ROPE = 100% | ℮𝛽 = 1.03 95% CI[1, 1.06] PD = 96.46% ROPE = 100% | ℮𝛽 = 1.03 95% CI[1, 1.06] PD = 98.88% ROPE = 100% | ℮𝛽 = 1.03 95% CI[1, 1.06] PD = 98.92% ROPE = 100% | ℮𝛽 = 1.02 95% CI[0.99, 1.05] PD = 88.79% ROPE = 100% | ℮𝛽 = 1.03 95% CI[1, 1.06] PD = 97.64% ROPE = 100% | ℮𝛽 = 1.03 95% CI[1, 1.06] PD = 98.21% ROPE = 100% | ℮𝛽 = 1.03 95% CI[1, 1.06] PD = 96.27% ROPE = 100% |
| **Gender** | ℮𝛽 = 0.69 95% CI[0.44, 1.07] PD = 95.02% ROPE = 9.11% | ℮𝛽 = 0.74 95% CI[0.47, 1.17] PD = 90.3% ROPE = 15.35% | ℮𝛽 = 0.75 95% CI[0.48, 1.15] PD = 90.74% ROPE = 16.43% | ℮𝛽 = 0.67 95% CI[0.43, 1.03] PD = 96.62% ROPE = 6.18% | ℮𝛽 = 0.74 95% CI[0.47, 1.17] PD = 89.98% ROPE = 15.82% | ℮𝛽 = 0.68 95% CI[0.43, 1.05] PD = 95.89% ROPE = 7.47% | ℮𝛽 = 0.64 95% CI[0.42, 0.99] PD = 97.68% ROPE = 3.77% | ℮𝛽 = 0.7 95% CI[0.46, 1.09] PD = 94.6% ROPE = 9.95% |
| **Group BD** | ℮𝛽 = 1.05 95% CI[0.58, 1.9] PD = 56.01% ROPE = 27.2% | ℮𝛽 = 1.08 95% CI[0.6, 1.96] PD = 60.68% ROPE = 26.76% | ℮𝛽 = 1.15 95% CI[0.64, 2.06] PD = 68.58% ROPE = 24.49% | ℮𝛽 = 1.14 95% CI[0.65, 2.01] PD = 67.82% ROPE = 25.65% | ℮𝛽 = 0.61 95% CI[0.33, 1.16] PD = 93.71% ROPE = 7.91% | ℮𝛽 = 1.08 95% CI[0.6, 1.96] PD = 60.02% ROPE = 26.35% | ℮𝛽 = 1.22 95% CI[0.68, 2.19] PD = 74.45% ROPE = 22.45% | ℮𝛽 = 1.09 95% CI[0.6, 1.96] PD = 61.3% ROPE = 27.27% |
| **Group MDD** | ℮𝛽 = 0.58 95% CI[0.31, 1.09] PD = 95.7% ROPE = 6.08% | ℮𝛽 = 0.68 95% CI[0.37, 1.22] PD = 89.68% ROPE = 12.72% | ℮𝛽 = 0.65 95% CI[0.36, 1.16] PD = 92.48% ROPE = 10.26% | ℮𝛽 = 0.66 95% CI[0.37, 1.18] PD = 92.22% ROPE = 10.15% | ℮𝛽 = 0.64 95% CI[0.35, 1.16] PD = 92.74% ROPE = 9.79% | ℮𝛽 = 0.6 95% CI[0.33, 1.09] PD = 95.16% ROPE = 7% | ℮𝛽 = 0.6 95% CI[0.33, 1.09] PD = 95.49% ROPE = 6.44% | ℮𝛽 = 0.67 95% CI[0.37, 1.21] PD = 90.55% ROPE = 11.95% |
| **Group SZ** | ℮𝛽 = 1.96 95% CI[1.04, 3.7] PD = 98.19% ROPE = 1.45% | ℮𝛽 = 2.04 95% CI[1.07, 3.91] PD = 98.47% ROPE = 0.79% | ℮𝛽 = 2.23 95% CI[1.18, 4.27] PD = 99.28% ROPE = 0% | ℮𝛽 = 2.19 95% CI[1.16, 4.07] PD = 99.28% ROPE = 0% | ℮𝛽 = 1.4 95% CI[0.71, 2.79] PD = 83.43% ROPE = 15.1% | ℮𝛽 = 2.03 95% CI[1.05, 3.96] PD = 98.14% ROPE = 1.2% | ℮𝛽 = 2.08 95% CI[1.08, 4.04] PD = 98.5% ROPE = 0.49% | ℮𝛽 = 2.08 95% CI[1.09, 3.96] PD = 98.59% ROPE = 0.37% |
| **FD** | ℮𝛽 = 0.85 95% CI[0.67, 1.08] PD = 90.81% ROPE = 29.27% | ℮𝛽 = 0.9 95% CI[0.72, 1.14] PD = 80.09% ROPE = 47.34% | ℮𝛽 = 0.93 95% CI[0.74, 1.18] PD = 73.01% ROPE = 55.71% | ℮𝛽 = 0.86 95% CI[0.69, 1.09] PD = 89.49% ROPE = 33.52% | ℮𝛽 = 0.96 95% CI[0.76, 1.21] PD = 62.37% ROPE = 60.05% | ℮𝛽 = 0.9 95% CI[0.71, 1.14] PD = 81.07% ROPE = 46.93% | ℮𝛽 = 0.94 95% CI[0.75, 1.18] PD = 69.44% ROPE = 58.48% | ℮𝛽 = 0.94 95% CI[0.75, 1.2] PD = 68.16% ROPE = 57.47% |
| **Measure** | ℮𝛽 = 1.14 95% CI[1.09, 1.18] PD = 100% ROPE = 7.56% | ℮𝛽 = 1.14 95% CI[1.09, 1.19] PD = 100% ROPE = 8.22% | ℮𝛽 = 1.14 95% CI[1.09, 1.18] PD = 100% ROPE = 8.07% | ℮𝛽 = 1.14 95% CI[1.09, 1.19] PD = 100% ROPE = 8.18% | ℮𝛽 = 1.14 95% CI[1.09, 1.2] PD = 100% ROPE = 8.78% | ℮𝛽 = 1.14 95% CI[1.09, 1.19] PD = 100% ROPE = 7.98% | ℮𝛽 = 1.14 95% CI[1.09, 1.18] PD = 100% ROPE = 7.5% | ℮𝛽 = 1.14 95% CI[1.09, 1.18] PD = 100% ROPE = 8.18% |
| **PC x Group BD** | ℮𝛽 = 0.69 95% CI[0.34, 1.41] PD = 84.65% ROPE = 13.65% | ℮𝛽 = 0.82 95% CI[0.43, 1.57] PD = 72.71% ROPE = 21.34% | ℮𝛽 = 0.88 95% CI[0.46, 1.65] PD = 65.44% ROPE = 24.45% | ℮𝛽 = 0.76 95% CI[0.42, 1.39] PD = 82.3% ROPE = 17.63% | ℮𝛽 = 0.99 95% CI[0.41, 2.38] PD = 50.92% ROPE = 18.22% | ℮𝛽 = 0.87 95% CI[0.44, 1.73] PD = 64.94% ROPE = 22.74% | ℮𝛽 = 0.64 95% CI[0.32, 1.24] PD = 90.5% ROPE = 10.36% | ℮𝛽 = 0.79 95% CI[0.44, 1.43] PD = 78.36% ROPE = 20.54% |
| **PC x Group MDD** | ℮𝛽 = 1.16 95% CI[0.6, 2.27] PD = 67.34% ROPE = 22.28% | ℮𝛽 = 1 95% CI[0.47, 2.16] PD = 50.11% ROPE = 21.39% | ℮𝛽 = 1.39 95% CI[0.79, 2.44] PD = 87.67% ROPE = 14.99% | ℮𝛽 = 1.31 95% CI[0.75, 2.33] PD = 82.04% ROPE = 18.68% | ℮𝛽 = 0.99 95% CI[0.47, 2.05] PD = 51.32% ROPE = 22.92% | ℮𝛽 = 1.27 95% CI[0.67, 2.38] PD = 77.73% ROPE = 18.93% | ℮𝛽 = 1.2 95% CI[0.66, 2.19] PD = 72.34% ROPE = 23.16% | ℮𝛽 = 0.82 95% CI[0.46, 1.46] PD = 76.12% ROPE = 21.71% |
| **PC x Group SZ** | ℮𝛽 = 0.93 95% CI[0.44, 2.02] PD = 57.08% ROPE = 20.34% | ℮𝛽 = 1 95% CI[0.49, 2.06] PD = 50.03% ROPE = 22.91% | ℮𝛽 = 1.42 95% CI[0.76, 2.63] PD = 86.33% ROPE = 14.59% | ℮𝛽 = 2 95% CI[1.07, 3.72] PD = 98.51% ROPE = 0.57% | ℮𝛽 = 0.87 95% CI[0.34, 2.24] PD = 61.51% ROPE = 16.88% | ℮𝛽 = 1.16 95% CI[0.66, 2.04] PD = 69.26% ROPE = 25.06% | ℮𝛽 = 0.86 95% CI[0.47, 1.56] PD = 69.24% ROPE = 23.39% | ℮𝛽 = 1.06 95% CI[0.55, 2.02] PD = 55.99% ROPE = 24.68% |
| **WN FZ Cor x Group BD** | ℮𝛽 = 0.62 95% CI[0.3, 1.3] PD = 89.84% ROPE = 9.78% | ℮𝛽 = 0.52 95% CI[0.26, 1.02] PD = 97.21% ROPE = 3.14% | ℮𝛽 = 0.58 95% CI[0.31, 1.06] PD = 96.17% ROPE = 5.16% | ℮𝛽 = 0.62 95% CI[0.33, 1.16] PD = 93.37% ROPE = 8.38% | ℮𝛽 = 0.76 95% CI[0.4, 1.46] PD = 79.01% ROPE = 17.81% | ℮𝛽 = 0.8 95% CI[0.44, 1.42] PD = 77.42% ROPE = 20.66% | ℮𝛽 = 0.9 95% CI[0.51, 1.57] PD = 63.73% ROPE = 27.39% | ℮𝛽 = 0.86 95% CI[0.49, 1.51] PD = 70.17% ROPE = 25.21% |
| **WN FZ Cor x Group MDD** | ℮𝛽 = 1.25 95% CI[0.66, 2.37] PD = 75.66% ROPE = 20.05% | ℮𝛽 = 0.95 95% CI[0.49, 1.87] PD = 55.4% ROPE = 24.46% | ℮𝛽 = 0.68 95% CI[0.37, 1.23] PD = 89.38% ROPE = 13.01% | ℮𝛽 = 1.18 95% CI[0.67, 2.07] PD = 72.64% ROPE = 24.17% | ℮𝛽 = 0.8 95% CI[0.41, 1.59] PD = 73.95% ROPE = 19.75% | ℮𝛽 = 1.19 95% CI[0.64, 2.24] PD = 70.62% ROPE = 22.3% | ℮𝛽 = 1.2 95% CI[0.66, 2.16] PD = 73.41% ROPE = 23.4% | ℮𝛽 = 1.36 95% CI[0.73, 2.53] PD = 82.86% ROPE = 16.82% |
| **WN FZ Cor x Group SZ** | ℮𝛽 = 0.79 95% CI[0.36, 1.75] PD = 71.86% ROPE = 17.64% | ℮𝛽 = 0.88 95% CI[0.43, 1.81] PD = 64.31% ROPE = 20.94% | ℮𝛽 = 0.64 95% CI[0.35, 1.18] PD = 92.45% ROPE = 9.95% | ℮𝛽 = 1.34 95% CI[0.67, 2.66] PD = 79.53% ROPE = 16.95% | ℮𝛽 = 0.37 95% CI[0.17, 0.8] PD = 99.31% ROPE = 0% | ℮𝛽 = 0.66 95% CI[0.36, 1.24] PD = 90.28% ROPE = 11.79% | ℮𝛽 = 0.83 95% CI[0.41, 1.64] PD = 69.39% ROPE = 20.72% | ℮𝛽 = 0.6 95% CI[0.27, 1.34] PD = 89.16% ROPE = 9.46% |

Supplemental Table 3. Spatial Extent aMAP Models

| **Predictor** | **DMN** | **FP** | **DA** | **VA** | **Sal** | **CO** | **DSM** | **VSM** |
| --- | --- | --- | --- | --- | --- | --- | --- | --- |
| **# of Vertices** | ℮𝛽 = 0.82 95% CI[0.47, 1.4] PD = 76.31% ROPE = 23.26% | ℮𝛽 = 0.88 95% CI[0.56, 1.38] PD = 70.78% ROPE = 30.24% | ℮𝛽 = 1.43 95% CI[0.88, 2.32] PD = 92.74% ROPE = 12.07% | ℮𝛽 = 1.46 95% CI[0.86, 2.46] PD = 92.22% ROPE = 11.84% | ℮𝛽 = 1.66 95% CI[1, 2.71] PD = 97.41% ROPE = 3.03% | ℮𝛽 = 1.06 95% CI[0.69, 1.63] PD = 61.29% ROPE = 36.13% | ℮𝛽 = 0.89 95% CI[0.54, 1.48] PD = 66.46% ROPE = 28.86% | ℮𝛽 = 1.23 95% CI[0.78, 1.95] PD = 81.38% ROPE = 24.59% |
| **Age** | ℮𝛽 = 1.03 95% CI[1, 1.07] PD = 96.78% ROPE = 100% | ℮𝛽 = 1.03 95% CI[1, 1.06] PD = 96.12% ROPE = 100% | ℮𝛽 = 1.03 95% CI[1, 1.07] PD = 97.53% ROPE = 100% | ℮𝛽 = 1.03 95% CI[1, 1.07] PD = 96.47% ROPE = 100% | ℮𝛽 = 1.04 95% CI[1.01, 1.07] PD = 98.99% ROPE = 100% | ℮𝛽 = 1.03 95% CI[1, 1.07] PD = 97.73% ROPE = 100% | ℮𝛽 = 1.03 95% CI[1, 1.07] PD = 97.5% ROPE = 100% | ℮𝛽 = 1.03 95% CI[1, 1.07] PD = 97.38% ROPE = 100% |
| **Gender** | ℮𝛽 = 0.75 95% CI[0.44, 1.27] PD = 85.99% ROPE = 17.72% | ℮𝛽 = 0.64 95% CI[0.38, 1.08] PD = 95.28% ROPE = 7.71% | ℮𝛽 = 0.68 95% CI[0.41, 1.16] PD = 92.36% ROPE = 11.51% | ℮𝛽 = 0.69 95% CI[0.42, 1.15] PD = 92.97% ROPE = 11.1% | ℮𝛽 = 0.69 95% CI[0.41, 1.16] PD = 92.04% ROPE = 12.35% | ℮𝛽 = 0.79 95% CI[0.47, 1.35] PD = 80.94% ROPE = 20.52% | ℮𝛽 = 0.73 95% CI[0.43, 1.24] PD = 87.82% ROPE = 15.34% | ℮𝛽 = 0.72 95% CI[0.43, 1.2] PD = 89.26% ROPE = 14.9% |
| **Group BD** | ℮𝛽 = 1.29 95% CI[0.66, 2.53] PD = 76.87% ROPE = 17.88% | ℮𝛽 = 1.31 95% CI[0.68, 2.58] PD = 79.13% ROPE = 17.96% | ℮𝛽 = 1.21 95% CI[0.61, 2.37] PD = 70.51% ROPE = 21.22% | ℮𝛽 = 1.17 95% CI[0.59, 2.27] PD = 67.86% ROPE = 21.41% | ℮𝛽 = 1.19 95% CI[0.6, 2.35] PD = 69.74% ROPE = 21.02% | ℮𝛽 = 1.37 95% CI[0.68, 2.74] PD = 81.66% ROPE = 16.23% | ℮𝛽 = 1.27 95% CI[0.63, 2.58] PD = 75.4% ROPE = 18.86% | ℮𝛽 = 1.36 95% CI[0.68, 2.73] PD = 80.59% ROPE = 16.97% |
| **Group MDD** | ℮𝛽 = 0.75 95% CI[0.39, 1.49] PD = 79.34% ROPE = 16.59% | ℮𝛽 = 0.68 95% CI[0.34, 1.34] PD = 86.26% ROPE = 13.14% | ℮𝛽 = 0.63 95% CI[0.3, 1.32] PD = 88.95% ROPE = 10.66% | ℮𝛽 = 0.71 95% CI[0.36, 1.41] PD = 84.01% ROPE = 14.54% | ℮𝛽 = 0.71 95% CI[0.35, 1.42] PD = 83.66% ROPE = 15.03% | ℮𝛽 = 0.76 95% CI[0.37, 1.52] PD = 78.32% ROPE = 17.16% | ℮𝛽 = 0.76 95% CI[0.38, 1.52] PD = 77.81% ROPE = 17.64% | ℮𝛽 = 0.78 95% CI[0.39, 1.58] PD = 75.33% ROPE = 18.49% |
| **Group SZ** | ℮𝛽 = 2.08 95% CI[0.98, 4.39] PD = 97.22% ROPE = 2.52% | ℮𝛽 = 2.21 95% CI[1.08, 4.59] PD = 98.74% ROPE = 0.34% | ℮𝛽 = 2.07 95% CI[0.99, 4.41] PD = 97.29% ROPE = 2.38% | ℮𝛽 = 2.19 95% CI[1.03, 4.66] PD = 98% ROPE = 1.38% | ℮𝛽 = 2.33 95% CI[1.12, 4.74] PD = 98.84% ROPE = 0% | ℮𝛽 = 2.02 95% CI[0.94, 4.33] PD = 96.41% ROPE = 3.51% | ℮𝛽 = 2.31 95% CI[1.11, 4.75] PD = 98.68% ROPE = 0% | ℮𝛽 = 2.48 95% CI[1.13, 5.35] PD = 98.84% ROPE = 0% |
| **FD** | ℮𝛽 = 0.87 95% CI[0.66, 1.15] PD = 83.44% ROPE = 37.72% | ℮𝛽 = 0.88 95% CI[0.67, 1.14] PD = 83.84% ROPE = 38.9% | ℮𝛽 = 0.94 95% CI[0.72, 1.24] PD = 66% ROPE = 51.03% | ℮𝛽 = 0.94 95% CI[0.71, 1.24] PD = 67.79% ROPE = 50.39% | ℮𝛽 = 0.93 95% CI[0.71, 1.22] PD = 70.79% ROPE = 48.68% | ℮𝛽 = 0.88 95% CI[0.67, 1.15] PD = 82.61% ROPE = 38.93% | ℮𝛽 = 0.93 95% CI[0.71, 1.21] PD = 71.1% ROPE = 49.74% | ℮𝛽 = 0.92 95% CI[0.7, 1.21] PD = 72.86% ROPE = 47.76% |
| **Measure** | ℮𝛽 = 1.06 95% CI[1.02, 1.11] PD = 99.78% ROPE = 98.89% | ℮𝛽 = 1.06 95% CI[1.02, 1.11] PD = 99.76% ROPE = 98.47% | ℮𝛽 = 1.06 95% CI[1.02, 1.11] PD = 99.72% ROPE = 98.77% | ℮𝛽 = 1.06 95% CI[1.02, 1.11] PD = 99.77% ROPE = 99.09% | ℮𝛽 = 1.06 95% CI[1.02, 1.11] PD = 99.72% ROPE = 98.62% | ℮𝛽 = 1.06 95% CI[1.02, 1.11] PD = 99.67% ROPE = 98.64% | ℮𝛽 = 1.06 95% CI[1.02, 1.11] PD = 99.79% ROPE = 98.85% | ℮𝛽 = 1.06 95% CI[1.02, 1.11] PD = 99.77% ROPE = 98.41% |
| **# of Vertices x Group BD** | ℮𝛽 = 1.09 95% CI[0.51, 2.4] PD = 58.63% ROPE = 20.97% | ℮𝛽 = 0.98 95% CI[0.49, 1.98] PD = 52.51% ROPE = 22.89% | ℮𝛽 = 0.7 95% CI[0.33, 1.46] PD = 82.91% ROPE = 14.47% | ℮𝛽 = 0.44 95% CI[0.19, 0.98] PD = 97.86% ROPE = 1.59% | ℮𝛽 = 0.38 95% CI[0.18, 0.8] PD = 99.48% ROPE = 0% | ℮𝛽 = 0.75 95% CI[0.38, 1.5] PD = 79.22% ROPE = 16.95% | ℮𝛽 = 1.08 95% CI[0.51, 2.36] PD = 57.99% ROPE = 20.89% | ℮𝛽 = 0.9 95% CI[0.46, 1.79] PD = 61.48% ROPE = 23.18% |
| **# of Vertices x Group MDD** | ℮𝛽 = 1.18 95% CI[0.58, 2.42] PD = 67.49% ROPE = 20.33% | ℮𝛽 = 0.65 95% CI[0.33, 1.27] PD = 89.14% ROPE = 11.62% | ℮𝛽 = 0.89 95% CI[0.45, 1.78] PD = 63.52% ROPE = 22.02% | ℮𝛽 = 0.8 95% CI[0.39, 1.66] PD = 72.67% ROPE = 18.79% | ℮𝛽 = 0.86 95% CI[0.45, 1.64] PD = 67.01% ROPE = 22.29% | ℮𝛽 = 0.88 95% CI[0.44, 1.77] PD = 63.48% ROPE = 22.43% | ℮𝛽 = 1.11 95% CI[0.55, 2.29] PD = 61.39% ROPE = 20.91% | ℮𝛽 = 0.84 95% CI[0.43, 1.63] PD = 69.54% ROPE = 20.89% |
| **# of Vertices x Group SZ** | ℮𝛽 = 0.91 95% CI[0.44, 1.88] PD = 60.89% ROPE = 22.05% | ℮𝛽 = 1.66 95% CI[0.84, 3.29] PD = 92.59% ROPE = 8.6% | ℮𝛽 = 0.53 95% CI[0.25, 1.15] PD = 94.51% ROPE = 5.98% | ℮𝛽 = 0.63 95% CI[0.31, 1.29] PD = 89.91% ROPE = 10.29% | ℮𝛽 = 0.61 95% CI[0.3, 1.21] PD = 92.02% ROPE = 8.66% | ℮𝛽 = 0.52 95% CI[0.26, 1.05] PD = 96.58% ROPE = 3.55% | ℮𝛽 = 1.37 95% CI[0.69, 2.69] PD = 81.36% ROPE = 16.21% | ℮𝛽 = 0.89 95% CI[0.4, 1.96] PD = 61.16% ROPE = 20.05% |

Supplemental Table 4. Spatial Extent cMAP Models

| **Predictor** | **DMN** | **FP** | **DA** | **VA** | **Sal** | **CO** | **DSM** | **VSM** |
| --- | --- | --- | --- | --- | --- | --- | --- | --- |
| **# of Vertices** | ℮𝛽 = 0.79 95% CI[0.49, 1.27] PD = 83.67% ROPE = 20.51% | ℮𝛽 = 0.9 95% CI[0.62, 1.32] PD = 70.7% ROPE = 37.07% | ℮𝛽 = 1.39 95% CI[0.92, 2.11] PD = 94.17% ROPE = 11.44% | ℮𝛽 = 1.35 95% CI[0.87, 2.08] PD = 91.44% ROPE = 15.55% | ℮𝛽 = 1.79 95% CI[1.17, 2.75] PD = 99.54% ROPE = 0% | ℮𝛽 = 1.08 95% CI[0.74, 1.57] PD = 65.18% ROPE = 39.5% | ℮𝛽 = 0.98 95% CI[0.64, 1.51] PD = 52.95% ROPE = 36.49% | ℮𝛽 = 1.13 95% CI[0.76, 1.68] PD = 72.08% ROPE = 34.26% |
| **Age** | ℮𝛽 = 1.03 95% CI[1, 1.06] PD = 97.2% ROPE = 100% | ℮𝛽 = 1.03 95% CI[1, 1.05] PD = 96.77% ROPE = 100% | ℮𝛽 = 1.03 95% CI[1, 1.06] PD = 98.11% ROPE = 100% | ℮𝛽 = 1.03 95% CI[1, 1.06] PD = 97% ROPE = 100% | ℮𝛽 = 1.04 95% CI[1.01, 1.07] PD = 99.49% ROPE = 100% | ℮𝛽 = 1.03 95% CI[1, 1.06] PD = 97.99% ROPE = 100% | ℮𝛽 = 1.03 95% CI[1, 1.06] PD = 97.52% ROPE = 100% | ℮𝛽 = 1.03 95% CI[1, 1.06] PD = 97.31% ROPE = 100% |
| **Gender** | ℮𝛽 = 0.73 95% CI[0.47, 1.13] PD = 91.74% ROPE = 14.8% | ℮𝛽 = 0.63 95% CI[0.41, 0.96] PD = 98.47% ROPE = 2.26% | ℮𝛽 = 0.66 95% CI[0.43, 1.02] PD = 96.89% ROPE = 5.57% | ℮𝛽 = 0.67 95% CI[0.44, 1.03] PD = 96.73% ROPE = 5.72% | ℮𝛽 = 0.67 95% CI[0.45, 1.02] PD = 97% ROPE = 5.72% | ℮𝛽 = 0.73 95% CI[0.47, 1.14] PD = 92.02% ROPE = 14.65% | ℮𝛽 = 0.71 95% CI[0.45, 1.1] PD = 93.92% ROPE = 11.55% | ℮𝛽 = 0.69 95% CI[0.45, 1.06] PD = 95.61% ROPE = 9.12% |
| **Group BD** | ℮𝛽 = 1.12 95% CI[0.63, 2] PD = 65.66% ROPE = 25.88% | ℮𝛽 = 1.13 95% CI[0.64, 1.99] PD = 66.83% ROPE = 26.39% | ℮𝛽 = 1.06 95% CI[0.59, 1.9] PD = 58.38% ROPE = 27.04% | ℮𝛽 = 1.03 95% CI[0.58, 1.84] PD = 53.08% ROPE = 28.47% | ℮𝛽 = 1.04 95% CI[0.6, 1.83] PD = 55.14% ROPE = 28.82% | ℮𝛽 = 1.14 95% CI[0.64, 2.05] PD = 67.51% ROPE = 25.11% | ℮𝛽 = 1.14 95% CI[0.64, 2.06] PD = 67.05% ROPE = 24.63% | ℮𝛽 = 1.15 95% CI[0.64, 2.05] PD = 67.5% ROPE = 24.84% |
| **Group MDD** | ℮𝛽 = 0.65 95% CI[0.37, 1.18] PD = 92.14% ROPE = 10.17% | ℮𝛽 = 0.59 95% CI[0.33, 1.04] PD = 96.59% ROPE = 4.96% | ℮𝛽 = 0.56 95% CI[0.31, 1.03] PD = 96.96% ROPE = 3.76% | ℮𝛽 = 0.62 95% CI[0.35, 1.1] PD = 94.79% ROPE = 7.48% | ℮𝛽 = 0.61 95% CI[0.35, 1.07] PD = 96% ROPE = 6.07% | ℮𝛽 = 0.67 95% CI[0.37, 1.2] PD = 91.22% ROPE = 11.3% | ℮𝛽 = 0.66 95% CI[0.37, 1.18] PD = 92.34% ROPE = 10.35% | ℮𝛽 = 0.66 95% CI[0.36, 1.22] PD = 90.86% ROPE = 11.19% |
| **Group SZ** | ℮𝛽 = 1.85 95% CI[0.98, 3.53] PD = 97.08% ROPE = 3.44% | ℮𝛽 = 1.93 95% CI[1.06, 3.53] PD = 98.29% ROPE = 0.98% | ℮𝛽 = 1.8 95% CI[0.97, 3.34] PD = 96.92% ROPE = 3.99% | ℮𝛽 = 1.92 95% CI[1.01, 3.66] PD = 97.63% ROPE = 2.2% | ℮𝛽 = 2 95% CI[1.1, 3.67] PD = 98.74% ROPE = 0.16% | ℮𝛽 = 1.85 95% CI[0.98, 3.44] PD = 97% ROPE = 3.28% | ℮𝛽 = 2.04 95% CI[1.11, 3.8] PD = 98.83% ROPE = 0% | ℮𝛽 = 2.01 95% CI[1.03, 3.9] PD = 97.92% ROPE = 1.87% |
| **FD** | ℮𝛽 = 0.87 95% CI[0.69, 1.09] PD = 88.14% ROPE = 36.39% | ℮𝛽 = 0.87 95% CI[0.7, 1.09] PD = 88.47% ROPE = 37.39% | ℮𝛽 = 0.93 95% CI[0.74, 1.17] PD = 72.73% ROPE = 55.32% | ℮𝛽 = 0.92 95% CI[0.73, 1.15] PD = 76.91% ROPE = 51.75% | ℮𝛽 = 0.92 95% CI[0.73, 1.17] PD = 74.94% ROPE = 52.74% | ℮𝛽 = 0.9 95% CI[0.71, 1.13] PD = 82.41% ROPE = 45.22% | ℮𝛽 = 0.91 95% CI[0.73, 1.14] PD = 79.21% ROPE = 49.71% | ℮𝛽 = 0.9 95% CI[0.72, 1.14] PD = 80.36% ROPE = 48.39% |
| **Measure** | ℮𝛽 = 1.14 95% CI[1.09, 1.18] PD = 100% ROPE = 7.66% | ℮𝛽 = 1.14 95% CI[1.09, 1.19] PD = 100% ROPE = 7.74% | ℮𝛽 = 1.14 95% CI[1.09, 1.19] PD = 100% ROPE = 7.2% | ℮𝛽 = 1.14 95% CI[1.09, 1.18] PD = 100% ROPE = 7.84% | ℮𝛽 = 1.14 95% CI[1.09, 1.18] PD = 100% ROPE = 8.09% | ℮𝛽 = 1.14 95% CI[1.09, 1.18] PD = 100% ROPE = 7.8% | ℮𝛽 = 1.14 95% CI[1.09, 1.19] PD = 100% ROPE = 8.47% | ℮𝛽 = 1.14 95% CI[1.09, 1.18] PD = 100% ROPE = 8.18% |
| **# of Vertices x Group BD** | ℮𝛽 = 1.06 95% CI[0.54, 2.1] PD = 57.29% ROPE = 23.19% | ℮𝛽 = 0.89 95% CI[0.5, 1.61] PD = 64.59% ROPE = 25.93% | ℮𝛽 = 0.73 95% CI[0.39, 1.37] PD = 83.03% ROPE = 16.26% | ℮𝛽 = 0.5 95% CI[0.25, 0.98] PD = 97.72% ROPE = 1.8% | ℮𝛽 = 0.37 95% CI[0.2, 0.68] PD = 99.92% ROPE = 0% | ℮𝛽 = 0.87 95% CI[0.49, 1.56] PD = 68.71% ROPE = 25.11% | ℮𝛽 = 1.16 95% CI[0.59, 2.24] PD = 67.37% ROPE = 22.88% | ℮𝛽 = 0.88 95% CI[0.48, 1.6] PD = 66.8% ROPE = 25.62% |
| **# of Vertices x Group MDD** | ℮𝛽 = 1.14 95% CI[0.61, 2.12] PD = 66.59% ROPE = 23.61% | ℮𝛽 = 0.67 95% CI[0.38, 1.17] PD = 92.05% ROPE = 11.34% | ℮𝛽 = 0.87 95% CI[0.49, 1.57] PD = 67.45% ROPE = 24.76% | ℮𝛽 = 1.09 95% CI[0.59, 2.01] PD = 61.46% ROPE = 26.24% | ℮𝛽 = 0.79 95% CI[0.46, 1.37] PD = 80.66% ROPE = 20.46% | ℮𝛽 = 0.94 95% CI[0.52, 1.74] PD = 57.67% ROPE = 26.72% | ℮𝛽 = 0.94 95% CI[0.51, 1.72] PD = 57.11% ROPE = 26% | ℮𝛽 = 0.98 95% CI[0.55, 1.73] PD = 53.14% ROPE = 28.47% |
| **# of Vertices x Group SZ** | ℮𝛽 = 1 95% CI[0.55, 1.83] PD = 50.66% ROPE = 26.66% | ℮𝛽 = 1.67 95% CI[0.94, 3] PD = 95.91% ROPE = 5.62% | ℮𝛽 = 0.51 95% CI[0.27, 0.96] PD = 98.13% ROPE = 1.32% | ℮𝛽 = 0.69 95% CI[0.39, 1.26] PD = 89.31% ROPE = 13.07% | ℮𝛽 = 0.53 95% CI[0.29, 0.95] PD = 98.28% ROPE = 1.36% | ℮𝛽 = 0.64 95% CI[0.35, 1.17] PD = 93.01% ROPE = 9.36% | ℮𝛽 = 1.28 95% CI[0.72, 2.27] PD = 79.86% ROPE = 20.25% | ℮𝛽 = 0.84 95% CI[0.42, 1.65] PD = 69.6% ROPE = 21.59% |

Supplemental Table 5. aMAP Secondary Between-Network Models

| **Predictor** | **VA & DMN** | **VA & FP** | **VA & DA** | **VA & Sal** | **VA & CO** | **VA & DSM** | **VA & VSM** |
| --- | --- | --- | --- | --- | --- | --- | --- |
| **BN FZ Cor** | ℮𝛽 = 1.94 95% CI[1.16, 3.23] PD = 99.44% ROPE = 0% | ℮𝛽 = 0.85 95% CI[0.54, 1.37] PD = 75.37% ROPE = 27.86% | ℮𝛽 = 0.62 95% CI[0.41, 0.94] PD = 98.7% ROPE = 1.23% | ℮𝛽 = 1.44 95% CI[0.85, 2.44] PD = 91.18% ROPE = 12.83% | ℮𝛽 = 1.3 95% CI[0.82, 2.05] PD = 86.54% ROPE = 19.62% | ℮𝛽 = 0.98 95% CI[0.59, 1.63] PD = 52.76% ROPE = 31.13% | ℮𝛽 = 1.57 95% CI[0.97, 2.54] PD = 96.81% ROPE = 5.61% |
| **Age** | ℮𝛽 = 1.03 95% CI[1, 1.07] PD = 97.94% ROPE = 100% | ℮𝛽 = 1.03 95% CI[1, 1.06] PD = 96.01% ROPE = 100% | ℮𝛽 = 1.03 95% CI[1, 1.07] PD = 97.77% ROPE = 100% | ℮𝛽 = 1.02 95% CI[0.99, 1.06] PD = 89.95% ROPE = 100% | ℮𝛽 = 1.03 95% CI[1, 1.06] PD = 95.88% ROPE = 100% | ℮𝛽 = 1.03 95% CI[1, 1.07] PD = 96.6% ROPE = 100% | ℮𝛽 = 1.03 95% CI[1, 1.06] PD = 95.4% ROPE = 100% |
| **Gender** | ℮𝛽 = 0.76 95% CI[0.46, 1.28] PD = 84.82% ROPE = 18.39% | ℮𝛽 = 0.69 95% CI[0.41, 1.15] PD = 92.19% ROPE = 12.32% | ℮𝛽 = 0.78 95% CI[0.47, 1.3] PD = 83.41% ROPE = 20.27% | ℮𝛽 = 0.71 95% CI[0.42, 1.18] PD = 90.38% ROPE = 13.55% | ℮𝛽 = 0.74 95% CI[0.44, 1.24] PD = 86.95% ROPE = 16.95% | ℮𝛽 = 0.72 95% CI[0.43, 1.21] PD = 88.96% ROPE = 15.1% | ℮𝛽 = 0.74 95% CI[0.44, 1.24] PD = 88.46% ROPE = 15.38% |
| **Group BD** | ℮𝛽 = 1.26 95% CI[0.65, 2.47] PD = 75.79% ROPE = 20.11% | ℮𝛽 = 1.34 95% CI[0.67, 2.64] PD = 80.94% ROPE = 16.05% | ℮𝛽 = 1.22 95% CI[0.62, 2.4] PD = 72.15% ROPE = 21.15% | ℮𝛽 = 0.65 95% CI[0.33, 1.28] PD = 89.74% ROPE = 11.28% | ℮𝛽 = 1.36 95% CI[0.68, 2.69] PD = 81.07% ROPE = 16.03% | ℮𝛽 = 1.31 95% CI[0.67, 2.55] PD = 78.22% ROPE = 18.09% | ℮𝛽 = 1.29 95% CI[0.67, 2.48] PD = 78.06% ROPE = 19.34% |
| **Group MDD** | ℮𝛽 = 0.81 95% CI[0.39, 1.64] PD = 71.58% ROPE = 19.71% | ℮𝛽 = 0.74 95% CI[0.37, 1.46] PD = 79.71% ROPE = 17.1% | ℮𝛽 = 0.73 95% CI[0.37, 1.42] PD = 82.74% ROPE = 15.72% | ℮𝛽 = 0.55 95% CI[0.28, 1.1] PD = 95.58% ROPE = 5.64% | ℮𝛽 = 0.79 95% CI[0.4, 1.58] PD = 74.42% ROPE = 19.29% | ℮𝛽 = 0.76 95% CI[0.38, 1.54] PD = 78.59% ROPE = 17.36% | ℮𝛽 = 0.71 95% CI[0.37, 1.4] PD = 83.89% ROPE = 14.69% |
| **Group SZ** | ℮𝛽 = 2.51 95% CI[1.21, 5.21] PD = 99.29% ROPE = 0% | ℮𝛽 = 2.28 95% CI[1.09, 4.77] PD = 98.62% ROPE = 0.22% | ℮𝛽 = 1.85 95% CI[0.87, 3.8] PD = 94.47% ROPE = 6.11% | ℮𝛽 = 1.07 95% CI[0.48, 2.38] PD = 56.51% ROPE = 19.73% | ℮𝛽 = 2.55 95% CI[1.19, 5.47] PD = 99.06% ROPE = 0% | ℮𝛽 = 2.41 95% CI[1.12, 5.23] PD = 98.72% ROPE = 0% | ℮𝛽 = 2 95% CI[0.96, 4.15] PD = 96.88% ROPE = 3.19% |
| **FD** | ℮𝛽 = 0.97 95% CI[0.74, 1.26] PD = 59.33% ROPE = 55.92% | ℮𝛽 = 0.91 95% CI[0.7, 1.19] PD = 75.02% ROPE = 47.81% | ℮𝛽 = 0.95 95% CI[0.73, 1.24] PD = 65.86% ROPE = 54.51% | ℮𝛽 = 0.98 95% CI[0.75, 1.29] PD = 54.95% ROPE = 54.57% | ℮𝛽 = 0.91 95% CI[0.7, 1.19] PD = 74.99% ROPE = 45.57% | ℮𝛽 = 0.9 95% CI[0.68, 1.2] PD = 77.06% ROPE = 42.72% | ℮𝛽 = 0.96 95% CI[0.74, 1.24] PD = 62.33% ROPE = 55.82% |
| **Measure** | ℮𝛽 = 1.06 95% CI[1.02, 1.11] PD = 99.73% ROPE = 98.89% | ℮𝛽 = 1.06 95% CI[1.02, 1.11] PD = 99.71% ROPE = 98.53% | ℮𝛽 = 1.06 95% CI[1.02, 1.11] PD = 99.76% ROPE = 98.56% | ℮𝛽 = 1.05 95% CI[1, 1.1] PD = 98.38% ROPE = 100% | ℮𝛽 = 1.06 95% CI[1.02, 1.11] PD = 99.64% ROPE = 98.74% | ℮𝛽 = 1.06 95% CI[1.02, 1.11] PD = 99.78% ROPE = 99.19% | ℮𝛽 = 1.06 95% CI[1.02, 1.11] PD = 99.78% ROPE = 98.72% |
| **BN FZ Cor x Group BD** | ℮𝛽 = 0.54 95% CI[0.29, 1.04] PD = 96.64% ROPE = 3.85% | ℮𝛽 = 1.5 95% CI[0.74, 3.03] PD = 86.87% ROPE = 12.55% | ℮𝛽 = 1.75 95% CI[0.96, 3.22] PD = 96.66% ROPE = 4.38% | ℮𝛽 = 0.72 95% CI[0.34, 1.53] PD = 80.42% ROPE = 14.93% | ℮𝛽 = 1.11 95% CI[0.53, 2.36] PD = 61.05% ROPE = 21.94% | ℮𝛽 = 0.88 95% CI[0.43, 1.88] PD = 63.1% ROPE = 19.75% | ℮𝛽 = 0.5 95% CI[0.25, 0.99] PD = 97.65% ROPE = 2.47% |
| **BN FZ Cor x Group MDD** | ℮𝛽 = 0.39 95% CI[0.19, 0.84] PD = 99.17% ROPE = 0% | ℮𝛽 = 1.28 95% CI[0.59, 2.74] PD = 73.71% ROPE = 17.11% | ℮𝛽 = 1.58 95% CI[0.78, 3.17] PD = 90.06% ROPE = 10.22% | ℮𝛽 = 0.55 95% CI[0.26, 1.15] PD = 94.3% ROPE = 6.66% | ℮𝛽 = 0.9 95% CI[0.45, 1.81] PD = 62.51% ROPE = 22.86% | ℮𝛽 = 0.9 95% CI[0.44, 1.86] PD = 61.84% ROPE = 22.03% | ℮𝛽 = 0.81 95% CI[0.41, 1.58] PD = 73.61% ROPE = 20.62% |
| **BN FZ Cor x Group SZ** | ℮𝛽 = 0.91 95% CI[0.42, 1.98] PD = 60.21% ROPE = 20.46% | ℮𝛽 = 1.76 95% CI[0.93, 3.32] PD = 95.83% ROPE = 5.62% | ℮𝛽 = 0.8 95% CI[0.36, 1.84] PD = 70.86% ROPE = 17.1% | ℮𝛽 = 0.69 95% CI[0.35, 1.38] PD = 85.78% ROPE = 13.24% | ℮𝛽 = 0.6 95% CI[0.29, 1.2] PD = 92.48% ROPE = 8.82% | ℮𝛽 = 1.14 95% CI[0.51, 2.5] PD = 63.06% ROPE = 19.4% | ℮𝛽 = 0.37 95% CI[0.18, 0.75] PD = 99.72% ROPE = 0% |

Supplemental Table 6. cMAP Secondary Between-Network Models

| **Predictor** | **DMN** | **FP** | **DA** | **Sal** | **CO** | **DSM** | **VSM** |
| --- | --- | --- | --- | --- | --- | --- | --- |
| **BN FZ Cor** | ℮𝛽 = 1.64 95% CI[1.06, 2.56] PD = 98.65% ROPE = 1.55% | ℮𝛽 = 0.88 95% CI[0.6, 1.31] PD = 73.16% ROPE = 33.26% | ℮𝛽 = 0.74 95% CI[0.52, 1.06] PD = 94.97% ROPE = 12.2% | ℮𝛽 = 1.47 95% CI[0.95, 2.28] PD = 95.86% ROPE = 7.95% | ℮𝛽 = 1.3 95% CI[0.86, 1.92] PD = 90.47% ROPE = 18.03% | ℮𝛽 = 0.98 95% CI[0.64, 1.5] PD = 52.88% ROPE = 37.24% | ℮𝛽 = 1.46 95% CI[0.97, 2.21] PD = 96.36% ROPE = 6.95% |
| **Age** | ℮𝛽 = 1.03 95% CI[1, 1.06] PD = 97.24% ROPE = 100% | ℮𝛽 = 1.03 95% CI[1, 1.05] PD = 97.04% ROPE = 100% | ℮𝛽 = 1.03 95% CI[1, 1.06] PD = 97.56% ROPE = 100% | ℮𝛽 = 1.02 95% CI[0.99, 1.05] PD = 92.6% ROPE = 100% | ℮𝛽 = 1.03 95% CI[1, 1.05] PD = 96.47% ROPE = 100% | ℮𝛽 = 1.03 95% CI[1, 1.06] PD = 97.42% ROPE = 100% | ℮𝛽 = 1.03 95% CI[1, 1.06] PD = 97.3% ROPE = 100% |
| **Gender** | ℮𝛽 = 0.75 95% CI[0.48, 1.15] PD = 90.83% ROPE = 15.87% | ℮𝛽 = 0.7 95% CI[0.44, 1.08] PD = 94.49% ROPE = 10.48% | ℮𝛽 = 0.76 95% CI[0.5, 1.16] PD = 89.82% ROPE = 17.88% | ℮𝛽 = 0.7 95% CI[0.46, 1.08] PD = 94.61% ROPE = 10.68% | ℮𝛽 = 0.73 95% CI[0.47, 1.12] PD = 92.84% ROPE = 13.63% | ℮𝛽 = 0.69 95% CI[0.45, 1.08] PD = 94.67% ROPE = 10.26% | ℮𝛽 = 0.69 95% CI[0.45, 1.06] PD = 95.17% ROPE = 8.81% |
| **Group BD** | ℮𝛽 = 1.13 95% CI[0.65, 1.97] PD = 66.48% ROPE = 26.12% | ℮𝛽 = 1.14 95% CI[0.65, 2.06] PD = 67.33% ROPE = 25.7% | ℮𝛽 = 1.07 95% CI[0.61, 1.87] PD = 59.61% ROPE = 27.95% | ℮𝛽 = 0.61 95% CI[0.34, 1.1] PD = 95.19% ROPE = 7.24% | ℮𝛽 = 1.17 95% CI[0.66, 2.06] PD = 70.49% ROPE = 24.9% | ℮𝛽 = 1.13 95% CI[0.64, 2] PD = 66.16% ROPE = 26.28% | ℮𝛽 = 1.13 95% CI[0.65, 2] PD = 67.14% ROPE = 26.29% |
| **Group MDD** | ℮𝛽 = 0.63 95% CI[0.34, 1.15] PD = 93.26% ROPE = 9.55% | ℮𝛽 = 0.66 95% CI[0.37, 1.21] PD = 91.31% ROPE = 10.09% | ℮𝛽 = 0.64 95% CI[0.36, 1.14] PD = 93.82% ROPE = 8.91% | ℮𝛽 = 0.48 95% CI[0.27, 0.86] PD = 99.36% ROPE = 0% | ℮𝛽 = 0.66 95% CI[0.36, 1.2] PD = 91.79% ROPE = 9.76% | ℮𝛽 = 0.66 95% CI[0.37, 1.17] PD = 92.38% ROPE = 10.42% | ℮𝛽 = 0.62 95% CI[0.36, 1.09] PD = 95.2% ROPE = 7.45% |
| **Group SZ** | ℮𝛽 = 2.11 95% CI[1.11, 3.96] PD = 98.86% ROPE = 0% | ℮𝛽 = 2 95% CI[1.06, 3.78] PD = 98.46% ROPE = 0.81% | ℮𝛽 = 1.68 95% CI[0.89, 3.12] PD = 94.51% ROPE = 7.01% | ℮𝛽 = 0.97 95% CI[0.49, 1.91] PD = 53.81% ROPE = 24.59% | ℮𝛽 = 2.14 95% CI[1.12, 4.04] PD = 98.89% ROPE = 0% | ℮𝛽 = 2.16 95% CI[1.11, 4.22] PD = 98.76% ROPE = 0% | ℮𝛽 = 1.79 95% CI[0.96, 3.29] PD = 96.74% ROPE = 3.94% |
| **FD** | ℮𝛽 = 0.94 95% CI[0.76, 1.18] PD = 69.06% ROPE = 59.33% | ℮𝛽 = 0.91 95% CI[0.73, 1.14] PD = 79.22% ROPE = 51.3% | ℮𝛽 = 0.92 95% CI[0.74, 1.16] PD = 76% ROPE = 52.39% | ℮𝛽 = 0.96 95% CI[0.77, 1.21] PD = 62.88% ROPE = 61.52% | ℮𝛽 = 0.89 95% CI[0.71, 1.13] PD = 84.11% ROPE = 43.38% | ℮𝛽 = 0.88 95% CI[0.7, 1.11] PD = 85.54% ROPE = 40.01% | ℮𝛽 = 0.95 95% CI[0.76, 1.18] PD = 67.94% ROPE = 61.78% |
| **Measure** | ℮𝛽 = 1.14 95% CI[1.09, 1.18] PD = 100% ROPE = 7.43% | ℮𝛽 = 1.14 95% CI[1.09, 1.19] PD = 100% ROPE = 7.8% | ℮𝛽 = 1.14 95% CI[1.09, 1.19] PD = 100% ROPE = 7.46% | ℮𝛽 = 1.13 95% CI[1.08, 1.19] PD = 100% ROPE = 13.41% | ℮𝛽 = 1.14 95% CI[1.09, 1.18] PD = 100% ROPE = 7.42% | ℮𝛽 = 1.14 95% CI[1.09, 1.19] PD = 100% ROPE = 7.62% | ℮𝛽 = 1.14 95% CI[1.09, 1.19] PD = 100% ROPE = 7.77% |
| **BN FZ Cor x Group BD** | ℮𝛽 = 0.74 95% CI[0.42, 1.3] PD = 85.28% ROPE = 17.17% | ℮𝛽 = 1.28 95% CI[0.71, 2.38] PD = 79.24% ROPE = 19.59% | ℮𝛽 = 1.36 95% CI[0.8, 2.3] PD = 87.61% ROPE = 15.56% | ℮𝛽 = 0.72 95% CI[0.38, 1.36] PD = 84.46% ROPE = 15.8% | ℮𝛽 = 1 95% CI[0.53, 1.91] PD = 50.46% ROPE = 26.02% | ℮𝛽 = 0.91 95% CI[0.49, 1.69] PD = 61.86% ROPE = 24.78% | ℮𝛽 = 0.53 95% CI[0.3, 0.95] PD = 98.38% ROPE = 0.98% |
| **BN FZ Cor x Group MDD** | ℮𝛽 = 0.64 95% CI[0.34, 1.22] PD = 91.23% ROPE = 10.22% | ℮𝛽 = 1.05 95% CI[0.54, 2.05] PD = 55.34% ROPE = 25.09% | ℮𝛽 = 1.08 95% CI[0.6, 1.97] PD = 60.27% ROPE = 25.11% | ℮𝛽 = 0.55 95% CI[0.3, 1.02] PD = 97.17% ROPE = 2.85% | ℮𝛽 = 0.78 95% CI[0.43, 1.42] PD = 79.39% ROPE = 19.67% | ℮𝛽 = 0.82 95% CI[0.46, 1.48] PD = 75.28% ROPE = 21.72% | ℮𝛽 = 0.89 95% CI[0.5, 1.56] PD = 65.21% ROPE = 26.56% |
| **BN FZ Cor x Group SZ** | ℮𝛽 = 0.85 95% CI[0.44, 1.65] PD = 68.03% ROPE = 22.03% | ℮𝛽 = 1.44 95% CI[0.84, 2.47] PD = 90.89% ROPE = 12.72% | ℮𝛽 = 0.77 95% CI[0.39, 1.54] PD = 77.2% ROPE = 17.86% | ℮𝛽 = 0.82 95% CI[0.46, 1.49] PD = 74.5% ROPE = 22.14% | ℮𝛽 = 0.69 95% CI[0.38, 1.26] PD = 88.85% ROPE = 13.43% | ℮𝛽 = 1.21 95% CI[0.63, 2.36] PD = 71.06% ROPE = 21.51% | ℮𝛽 = 0.46 95% CI[0.25, 0.86] PD = 99.19% ROPE = 0% |
